# Circulating Proteins Associated with Response and Resistance to Neoadjuvant Chemotherapy in HER2-Positive Breast Cancer

**DOI:** 10.3390/cancers14041087

**Published:** 2022-02-21

**Authors:** María del Pilar Chantada-Vázquez, Mercedes Conde-Amboage, Lucía Graña-López, Sergio Vázquez-Estévez, Susana B. Bravo, Cristina Núñez

**Affiliations:** 1 Research Unit, Lucus Augusti University Hospital (HULA), Servizo Galego de Saúde (SERGAS), 27002 Lugo, Spain; mariadelpilarchantadavazquez@gmail.com; 2 Proteomic Unit, Health Research Institute of Santiago de Compostela (IDIS), University Clinical Hospital of Santiago de Compostela (CHUS), 15706 Santiago de Compostela, Spain; 3 Models of Optimization Decision, Statistics and Applications Research Group (MODESTYA), Department of Statistics, Mathematical Analysis and Optimization, Universidade de Santiago de Compostela, 15782 Santiago de Compostela, Spain; mercedes.amboage@usc.es; 4 CITMAga, 15782 Santiago de Compostela, Spain; 5 Breast Pathology Group, Lucus Augusti University Hospital (HULA)-IDIS, Servizo Galego de Saúde (SERGAS), 27002 Lugo, Spain; lucia.grana.lopez@sergas.es; 6 Radiology Department, Lucus Augusti University Hospital (HULA), Servizo Galego de Saúde (SERGAS), 27002 Lugo, Spain; 7 Oncology Division, Lucus Augusti University Hospital (HULA), Servizo Galego de Saúde (SERGAS), 27002 Lugo, Spain; sergio.vazquez.estevez@sergas.es

**Keywords:** breast cancer, HER2-positive, neoadjuvant, predictive, biomarkers, proteomics

## Abstract

**Simple Summary:**

The goal of this study was to find circulating proteins that can be easily sampled and incorporated into a clinical setting to improve predictive treatment response in HER2-positive breast cancer patients receiving neoadjuvant chemotherapy. We looked for potential biomarkers in serum, which we identified using two proteomics techniques: qualitative LC-MS/MS and a quantitative assay that assessed protein expression between responders and non-responders HER2-positive breast cancer patients to neoadjuvant chemotherapy.

**Abstract:**

Despite the increasing use of neoadjuvant chemotherapy (NAC) in HER2-positive breast cancer (BC) patients, the clinical problem of predicting individual treatment response remains unanswered. Furthermore, the use of ineffective chemotherapeutic regimens should be avoided. Serum biomarker levels are being studied more and more for their ability to predict therapy response and aid in the development of personalized treatment regimens. This study aims to identify effective protein networks and biomarkers to predict response to NAC in HER2-positive BC patients through an exhaustive large-scale LC-MS/MS-based qualitative and quantitative proteomic profiling of serum samples from responders and non-responders. Serum samples from HER2-positive BC patients were collected before NAC and were processed by three methods (with and without nanoparticles). The qualitative analysis revealed differences in the proteomic profiles between responders and non-responders, mainly in proteins implicated in the complement and coagulation cascades and apolipoproteins. Qualitative analysis confirmed that three proteins (AFM, SERPINA1, APOD) were correlated with NAC resistance. In this study, we show that serum biomarker profiles can predict treatment response and outcome in the neoadjuvant setting. If these findings are further developed, they will be of significant clinical utility in the design of treatment regimens for individual BC patients.

## 1. Introduction

For an increasing number of breast cancer (BC) patients, neoadjuvant chemotherapy (NAC), or the administration of chemotherapy and other agents before surgery, is the first line of treatment [1]. Among BC subtypes, those with human epidermal growth factor receptor 2 (HER2) positivity have been shown to have a tumor biology and a greater likelihood of pathologic complete response (pCR) when treated with NAC. Particularly, in HER2-positive breast tumors, neoadjuvant trastuzumab used in combination with standard chemotherapy can induce a 30% pCR rate [2,3,4]. 

With modern regimens incorporating dual anti-HER2 therapy with trastuzumab and pertuzumab, such as those used in the NeoSphere [5] and Tryphaena [6] trials, pCR rates range from 46% to 66%. This pCR rate was confirmed in the GeparSepto [7], KRISTINE [8], Symphony [9], and BERENICE [10] studies. 

pCR is a surrogate marker for evaluating response to HER2-targeted NAC and a prognostic marker for survival in many studies, but pCR is not achieved in all patients. In the neoadjuvant setting, there are currently no clinically established pre-treatment predictors of response. Given the physical [11] and financial [12] costs of treatment, predictive indicators of HER2-targeted NAC response would be extremely useful in identifying patients who will benefit the most from neoadjuvant therapy and guiding the selection of the most effective techniques from the start [13].

A growing body of research implies that chemotherapy success is linked to the tumor’s molecular profile [14], as well as the host response to therapy [15]. In pre-therapeutic biopsies of tumor and adjacent host tissue, profiling methods have enabled genome- or proteome-wide searches for predictive and prognostic biomarkers [16,17,18,19]. 

For example, bulk gene expression profiling of pre-treatment tissue samples has identified tumor characteristics (HER2-enriched intrinsic subtype, HER2 expression levels, ESR1 expression levels [20,21,22,23,24], and microenvironmental characteristics (increased immune infiltration [22,24,25,26,27]) that are linked to the response to HER2-targeted therapy in the neoadjuvant setting. 

As examples of tissue proteomic profiling studies, K.L. McNamara et al. [28] used a multiplex spatial proteomic biomarker to demonstrate substantial stratification of sensitive cancers early during neoadjuvant HER2-targeted therapy, with implications for tailoring subsequent therapy. M.H. Haugen et al. [29] determined by reverse-phase protein arrays (RPPA) a nine-protein signature score in tumor samples able to predict the response to neoadjuvant treatment with bevacizumab in combination with chemotherapy in HER2-negative BC. Y.-C. Chen et al. [30] found that the carboxyl-terminal modulator protein (CTMP) was a predictive biomarker for trastuzumab resistance in HER2-positive BC patients.

All these studies concentrated on tissue. However, tissue is not the ideal source of material for early diagnostic indicators because invasive sampling processes can injure or kill the organism being studied [31]. Recently, there has been a surge in interest in the identification and description of cancer diagnosis using noninvasive surrogate markers. Biomarkers in liquid biopsy have the following advantages: (a) they can detect a missing invasion; (b) they can be performed in ambulatory settings; (c) they can be checked repeatedly; and (d) they can be used for disease diagnosis and progression monitoring [32]. 

In this way, the variance of noncoding RNAs in serum was linked to clinical characteristics and progression, as well as the survival time of HER2-positive BC patients receiving trastuzumab-based therapy, according to several studies [33,34,35,36]. It was also discovered that HER2-positive BC patients with a serum HER2 ECD of more than 15 ng/mL [37,38], a greater carbonic anhydrase (CAIX) [38], or metalloproteinases [39] had shorter progression-free survival (PFS). In addition, increased fibrinogen levels in plasma were linked to a poor response to trastuzumab treatment in HER2-positive BC [40]. 

The diagnosis [41], monitoring [42], progression [43], and time prediction [44] of various malignancies have all been detailed using global quantitative proteomics analysis of blood samples to uncover possible biomarkers of the disease. However, to the authors’ knowledge, only one large-scale liquid chromatography-tandem mass spectrometry (LC–MS/MS)-based quantitative proteomic study was recently developed to find biomarkers of trastuzumab-based therapy resistance from the serum of HER2-positive BC cases [45]. 

Nanomaterials have been incorporated into the science of proteomics to create nanoproteomics, a new and fast-expanding research topic [46]. It is well understood that dispersing a nanomaterial in physiological fluid results in the formation of a protein shell known as a “protein corona” (PC). Disease-related biomarkers account for fewer than 1% of serum proteins. As a result of the PC formation, nanoparticles could act as sorbent materials of low-abundance proteins in serum samples before the biomarker identification via mass spectrometry (MS) analysis [47,48,49,50,51,52]. Characterization of the PC surrounding NPs has distinct advantages over sole proteomic approaches and increases the likelihood of identifying novel molecular biomarkers [53]. Thus, otherwise undetectable changes in the serum protein concentration of HER2-positive BC patients before NAC could be detected by analyzing the PC composition. 

**Figure 1:**
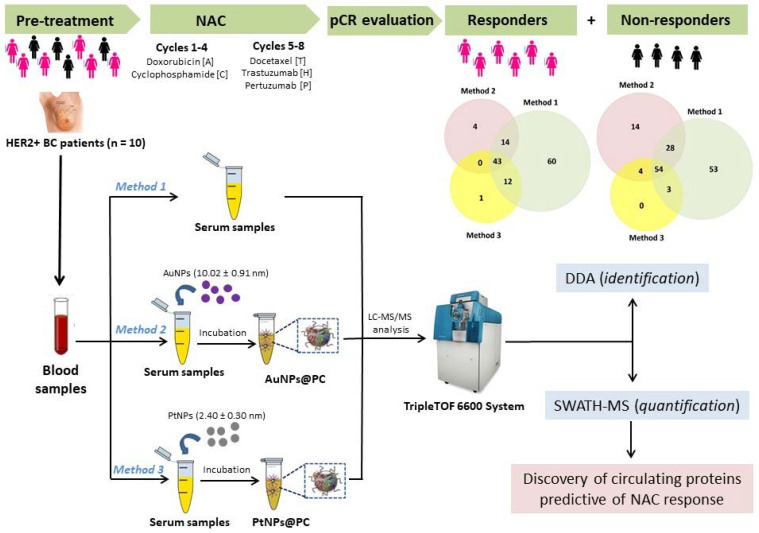
A schematic diagram of experimental workflow.

Particularly, the unique features of gold (AuNPs) [54] and platinum nanoparticles (PtNPs) [55] make them suitable sorbent nanomaterials with important biomedical applications. In the present study, the interaction of AuNPs (10.02 ± 0.91 nm) and PtNPs (2.40 ± 0.30 nm) with the sera of HER2-positive BC patients obtained before NAC allowed the pre-concentration of the low-abundance proteins through the PC formation. Then, an exhaustive large-scale LC-MS/MS-based qualitative and quantitative proteomic analysis of the PCs and the crude sera samples (without NPs) was carried out to explore potential circulating protein biomarkers useful to predict the therapeutic response of HER2-positive BC patients treated with NAC (see Figure 1). The results of this study could represent a useful tool to support clinical decision-making in HER2-positive BC patients. 

## 2. Materials and Methods

### 2.1. Patient Study Group 

Ten patients with pathologically proven HER2 overexpressing BC diagnosed at HULA, who had undergone breast MRI for monitoring the response to NAC from June 2017 to December 2018 were enrolled in the present study. The clinical characteristics of the patient study group are presented in Table 1. See inclusion and exclusion criteria in the Supplemental Material (Annex 1).

Patients received a combination of NAC with AC regimen: doxorubicin ([A] 60 mg/m^2^ iv), cyclophosphamide ([C], 600 mg/m^2^ iv) on day one every three weeks for four cycles. Subsequently, they were administered the combination with THP scheme: docetaxel ([T] 75 mg/m^2^ iv), trastuzumab ([H], at the loading dose of 8 mg/kg iv and then 6 mg/kg iv), and pertuzumab ([P], at the loading dose of 840 mg/m^2^ iv, then 420 mg/m^2^ iv), on day one every three weeks for four cycles. 

After NAC, all patients underwent surgery and surgical specimens were examined by a single pathologist, blinded to the study, who assessed the treatment response according to TNM [56]. Pathologic complete response (pCR) was defined as no residual invasive disease in both breast and axillary lymph nodes after NAC (ypT0/is, ypN0) at surgical resection.

After surgery, if invasive residual disease was detected, patients received adjuvant treatment with TDM-1 (3.6 mg/kg iv) on day 1 every 3 weeks for 14 cycles. If there was no presence of invasive residual disease, adjuvant treatment was with Trastuzumab at the doses previously described for 14 cycles.

### 2.2. Collection and Storage of Blood Serum

Blood samples from the ten HER2 overexpressing BC patients were obtained before NAC treatment. Eight milliliters of peripheral blood were collected in sterile VACUETTE^®^ Serum Clot Activator Tubes. Blood was allowed to coagulate for up to 15 min at room temperature. Then, samples were centrifuged at 1800× *g* for 5 min at 4 °C, and serum samples were aliquoted and stored at −80 °C for the proteomic analysis. Before taking part in the study, all participants signed a written consent form.

### 2.3. Chemicals and Reagents 

All reagents and solvents used were HPLC-grade or higher. Acrylamide/bis-acrylamide 30% solution (37.5:1), β-mercaptoethanol (molecular biology grade), chloroplatinic acid hexahydrate (≥37.50% Pt basis), Coomassie Brilliant Blue R250 (CBB), DL-dithiothreitol (DTT, 99%,), glycerol (86%–88%), iodoacetamide (IAA, 99%), sodium borohydride (99%), sodium citrate tribasic dihydrate (99%), sodium carbonate (99%), tris-base, trifluoroacetic acid (99%), trypsin from bovine pancreas, and the Sigma Marker wide range 6.5–200 KDa were purchased from Merck (Hohen-Brunn, Germany). Formaldehyde for molecular biology (36.5–38% in H_2_O) and sodium dodecyl sulfate (SDS) were purchased from Panreac (Barcelona, Spain). Bromophenol-blue was purchased from Riedel-de Haen (Seelze, Germany). Hydrogen tetrachloroaurate (III) hydrate (99.9%-Au) (49% Au) at 10% *w*/*v* was purchased from Strem Chemicals (Newburyport, MA, USA). Ammonium bicarbonate (AMBIC, 99.5%) and formic acid (95%) were purchased from Fluka (Steinheim, Germany).

### 2.4. Synthesis of Inorganic Nanoparticles

AuNPs (10.02 ± 0.91 nm) and PtNPs (2.40 ± 0.30 nm) were prepared following a citrate reduction method in an aqueous solution previously reported by our group [47]. See synthesis and characterization details in the Supplemental Material (Annex 2, Figures S1 and S2).

### 2.5. Instrumentation

For sodium dodecyl sulfate-polyacrylamide gel electrophoresis (SDS-PAGE) protein separation, a Power Pac Basic power supply from Bio-Rad (Hercules, CA, USA) was used. Protein quantification was accomplished by measuring absorbance at 280 nm with a Thermo Fisher Scientific Qubit^TM^ 4 Quantitation Starter Kit.

### 2.6. Depletion of Multiple High-Abundance Proteins in Serum Samples

A Miller-GP^®^ Filter Unit (Millipore) with a size of 0.22 μm was used to filter human serum samples. Six aliquots of human serum (30 µL) from each patient were depleted with dithiothreitol (DTT), according to the protocol described by Warder et al. [57,58]. Fresh DTT 500 mM (3.3 µL) in milli-Q water was quickly mixed and vortexed with 30 µL of human serum. The samples were then incubated at room temperature for 60 min until a viscous white precipitate formed, followed by 20-min centrifugation at 18,840× *g*. Before the protein fractionation, the supernatants were transferred to a clean tube.

### 2.7. Isolation, Fractionation and Digestion of Low-Abundance Proteins 

After the depletion of high-abundance proteins with DTT, *n* = 6 aliquots of serum from each patient were treated following three different approaches for the analysis of low-abundance proteins (candidate biomarkers).

**Method 1:***n* = 2 aliquots were transferred directly to a 10% SDS-PAGE gel to initiate whole protein concentration/separation.

**Method 2:***n* = 2 aliquots were alkylated with iodoacetic acid (IAA) at room temperature for 45 min and then protected from light. After protein reduction and alkylation, 75 μL of gold nanoparticles (AuNPs, 10.02 ± 0.91 nm) were added to each different aliquot, followed by the addition of 40 μL of citrate/citric acid buffer to a final pH of 5.8. The NPs–serum solutions were then incubated for 30 min at 37 °C with shaking in a thermostatic bath. Pellets were collected by centrifugation at 18,840× *g* for 30 min. Pellets containing proteins bound to nanoparticles were washed three times with a 25 L citrate/citric acid buffer before being centrifuged at 18,840× *g* for 30 min to remove unbound proteins.

**Method 3:** two aliquots (*n* = 2) were alkylated and incubated with platinum nanoparticles (PtNPs, 2.40 ± 0.30 nm), following the steps described in method 2 but increasing the centrifugation to 24,610× *g.*

Pellets from **method 2** and **method 3** were reconstituted and loaded on a 10% SDS-PAGE gel to initiate whole protein separation. The gel was stained, and the band was exscinded and submitted to an in-gel tryptic digestion method previously reported by our group [48,49,50,51,59].

### 2.8. Qualitative Proteomic Analysis by Mass Spectrometry (LC-MS/MS): Identification by Data-dependent Acquisition (DDA)

Following the conditions previously reported by our group [48,49,50,52], digested peptides of each sample were separated by reverse-phase chromatography (RPC), and protein identification was revealed using a nanoLC 400 system (Eksigent Tech., Dublin, CA, USA) coupled to a high-speed Triple TOF 6600 mass spectrometer (AB Sciex, Toronto, ON, Canada) with a microflow source. The analytical column used was a silica-based reversed phase column YMC-TRIART C18 150 × 0.30 mm, 3 mm particle size and 120 Å pore size (YMC Technologies, Teknokroma). The trap column was a YMC-TRIART C18 (YMC Technologies, Teknokroma) with a 3 mm particle size and 120 Å pore size, switched on-line with the analytical column. The loading pump delivered a solution of 0.1% formic acid in water at 10 μL/min. The micro-pump provided a flowrate of 5 μL/min and was operated under gradient elution conditions, using 0.1% formic acid in water as mobile phase A, and 0.1% formic acid in acetonitrile as mobile phase B. Peptides were separated using a 25 min gradient ranging from 2% to 90% mobile phase B (mobile phase A: 2% acetonitrile, 0.1% formic acid; mobile phase B: 100% acetonitrile, 0.1% formic acid). Injection volume was 4 μL.

Data acquisition was carried out in a TripleTOF 6600 System (SCIEX, Foster City, CA, USA) using a data dependent workflow. Source and interface conditions were as follows: ion spray voltage floating (ISVF) 5500 V, curtain gas (CUR) 25, collision energy (CE) 10, and ion source gas 1 (GS1) 25. The instrument was operated with Analyst TF 1.7.1 software (SCIEX, USA). Switching criteria was set to ions greater than mass to charge ratio (*m*/*z*) 350 and smaller than *m*/*z* 1400 with a charge state of 2–5, mass tolerance 250 ppm, and an abundance threshold of more than 200 counts (cps). Former target ions were excluded for 15 s. The instrument was automatically calibrated every 4 h using external calibrant tryptic peptides from PepcalMix (Sciex).

After the MS/MS acquisition, data files were processed using ProteinPilot^TM^ 5.0.1 software (version 5.0.1; AB Sciex) which uses the algorithm Paragon^TM^ for database search and Progroup^TM^ for data grouping. Data were searched using a human specific Uniprot database. A false discovery rate was performed using a non-lineal fitting method displaying only those results that reported a 1% Global false discovery rate or better [60,61].

### 2.9. Quantitative Proteomic Analysis by Sequential Window Acquisition of All Theoretical Mass Spectrometry (SWATH-MS) 

Samples were analyzed following the methodology previously described by our group [48,50,52]. Briefly, two biological replicates of responders and non-responders samples were used to get extensive quantitative data by label-free SWATH-MS analysis. Peptides of all samples were analyzed with a micro-LC system Ekspert nLC425 (Eksigen, Dublin, CA, USA) coupled to a hybrid quadrupole-TOF mass spectrometer Triple TOF 6600 (Sciex, Redwood City, CA, USA). One of the first steps was the construction of the MS/MS spectral libraries. For that purpose, peptide solutions were analyzed by a shotgun data-dependent acquisition (DDA) approach by micro-LC-MS/MS. For spectral alignment and peak extraction the Peakview software (version 2.2; AB Sciex) was employed using the SWATH Acquisition MicroApp (version 2.0). Parameters used were: number of fragments = 7, number of peptides = 10, peptide confidence = 95%, XIC width = 30 ppm, XIC extraction window = 5 min. Exportation of the SWATH file to the MarkerView software (version 1.3.1; AB Sciex) allowed the quantitative analysis of ions, peptides, and proteins in the different samples. As output result, the summed intensity of ions for the peptide, summed intensity of the peptides for protein, and Area under Curve (AUC) of the ions were provided. Both test sets (responders and non-responders) were compared to generate fold change ratios. For protein quantitation, only peptides with a False Discovery Rate (FDR) below 1% were considered. To compare the data across samples, an unsupervised multivariate statistical analysis using Principal Component Analysis (PCA) was performed. The mean area sums of all the transitions derived for each protein in each sample will be used in a Student’s *t*-test to determine how well each variable distinguishes the two groups, which will be presented as a *p*-value. For each library, its set of differentially expressed proteins (*p*-value < 0.05) with a FCh > 1.1 or <0.8 was selected.

### 2.10. Protein Functional Interaction Network Analysis 

The tool STRING v.10.0 database was used to analyze protein networks of functional interactions, incorporating direct (physical) and indirect protein–protein interactions (PPI) (http://string-db.org (accessed on 13 July 2021)) [62]. 

### 2.11. Statistical Analysis

All analyses were performed using SPSS Statistics 21.0 software (IBM, Armonk, NY, USA). As appropriate, a one- or two-tailed Student’s *t*-test or a Mann–Whitney U test was used. The statistical significance was defined as *p* < 0.05. Volcano plots and box plots were created with GraphPad Prism (GraphPad Software, San Diego, CA, USA) and a heat map was performed using http://www.heatmapper.ca/expression (accessed on 1 December 2021).

### 2.12. Development of the Classifiers

Different logistic regression models were adjusted to the data to determine the power of the different proteins to classify samples in the two categories that are considered. Associated with this model, receiver-operating characteristic (ROC) curves were generated and the area under the ROC curve (AUC), and the sensitivity and specificity at the “optimal” cutoff point for discrimination purposes between groups were obtained. All ROC analyses were performed using the R “pROC” package [63], where the optimal cutoff was selected so that the distance to the identity (diagonal) line was maximized, that is, max (sensitivities + specificities). AUCs 95% CIs were computed using Delong’s method [64] and the 95% CIs of the sensitivity and specificity values were computed with bootstrap resampling, see [65] for details. Moreover, to assess the robustness of the different proteins to classify, the “leave-one-out” cross-validation procedure was performed by applying to each measurement in the logistic regression model adjusted to the remaining sample of the dataset and afterwards performing the usual ROC analysis.

## 3. Results

### 3.1. Clinicopathological Features of Patients

In the present study, we collected serum from 10 primary HER2-positive BC cases receiving NAC at Hospital Universitario Lucus Augusti (HULA) with the experimental protocol approved by the Ethics Committee of this center. Blood samples were collected prior to patients receiving NAC and the patient characteristics were presented in Table 1. All patients’ HER2-positive status was determined using immunohistochemistry or fluorescence in situ hybridization coloration. Among all the patients, six patients acquiring a pCR were regarded as NAC response or “responders”, while four patients still had disease progression, defined as NAC resistance or “non-responders”, after neoadjuvant chemotherapy.

### 3.2. Proteomic Discovery Using the DDA Approach

Serum samples from responders (*n* = 6) and non-responders (*n* = 4) cases were then analyzed by mass spectrometry (LC-MS/MS) for protein identification to discover serum fingerprint proteins related to the NAC resistance/response in a parallel manner. 

Previous studies have shown that there are thousands of large dynamic proteins in serum, ranging from extremely low-abundance proteins to high-abundance proteins, with the latter being able to mask the identification and determination of the low-abundance proteins following quantitative analysis [66]. 

First, we performed high-abundance proteins depletion with dithiothreitol (DTT) following the protocol previously published by our group [48,49,50,51]. From each patient, six aliquots of serum were taken and depleted with DTT and were treated with three different methods for the analysis of low-abundance proteins:

**Method 1:** Two aliquots were loaded directly onto a 10% SDS-PAGE gel to initiate whole protein concentration. Then, the gel was stained, the bands were excised and submitted to in-gel tryptic digestion (see experimental Section 2.7) before the proteomic analysis. 

**Method 2:** Two aliquots were incubated with AuNPs (10.02 ± 0.91 nm). 

**Method 3:** Two aliquots were incubated with PtNPs (2.40 ± 0.30 nm).

In **method 2** and **method 3**, the protein concentration was promoted through the formation of the ex vivo protein corona around AuNPs and PtNPs. After that, proteins were separated from the NPs surface by gel electrophoresis following the procedure described in **method 1**. 

**Figure 2:**
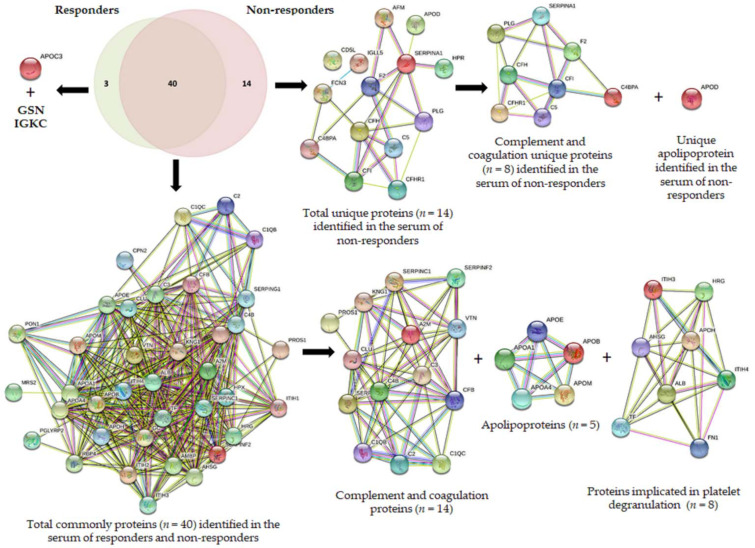
Venn diagram showing the number of proteins identified in the serum samples belonging to HER2-positive BC patients that were obtained before NAC. These patients showed a different response after the NAC treatment: responders (*n* = 6), non-responders (*n* = 4). Clusters found in the protein–protein interaction network map of the 43 and 54 genes encoded differentially proteins identified in serum samples from responders and non-responders before NAC, respectively. Based on the STRING database, a cluster of 14 proteins implicated in the complement and coagulation cascades were commonly identified in the serum of responders and non-responders (C1QB, C1QC, C2, C3, C4B, CFB, SERPINC1, SERPINF2, SERPING1, PROS1, VTN, A2M, CLU, KNG1), and a cluster of 8 proteins (C4BPA, C5, CFI, CFH, CFHR1, SERPINA1, F2, PLG) were specific to the non-responder’s group. Based on the STRING database, a cluster of 5 apolipoproteins were commonly identified in the serum of responders and non-responders (APOA1, APOA4, APOB, APOE, APOM), and APOC3 and APOD were specific of the responders and non-responders’ groups, respectively. A cluster of 8 proteins implicated in platelet degranulation (commonly identified in the serum of responders and non-responders (ALB, AHSG, APOH, FN1, HRG, ITIH3, ITIH4, TF) were also identified.

As Table 2 shows, a total of 129, 61, and 56 proteins were identified by LC-MS/MS in all serum samples from responders (*n* = 6), and 138, 100, and 61 proteins were identified in all serum samples from non-responders (*n* = 4) with the sample treatment **method 1**, **method 2**, and **method 3**, respectively (see Table 2 and Tables S1–S3). After comparing the results obtained by the three methods, 43 and 54 proteins were commonly identified in responders and non-responders cases, respectively (see Table 2, Tables S4 and S5). From them, 40 proteins were commonly detected in responders and non-responders, three proteins were only identified in responders and 14 proteins in non-responders (see Table 3 and Figure 2).

To interpret global changes in the serum proteome linked to NAC response/resistance in HER2-positive BC patients, the 43 and 54 proteins commonly identified by the three methods in the serum of responders and non-responders before NAC, respectively, were analyzed using the STRING software (see Figure 2). The analysis revealed that 14 from 43 proteins identified in responders and 22 from 54 proteins identified in non-responders were associated with complement and coagulation cascades. From them, 14 proteins were commonly identified in the serum of both groups, and they could be subdivided into:

(a) complements: complement C1q subcomponent subunit B (C1QB), complement C1q subcomponent subunit C (C1QC), complement C2 (C2), complement C3 (C3), complement C4-B (C4B), complement factor B (CFB);
(b) serine protease related proteins: antithrombin-III (SERPINC1), alpha-2-antiplasmin (SERPINF2), plasma protease C1 inhibitor (SERPING1);
(c) vitamin K-dependent proteins: vitamin K-dependent protein S (PROS1), and
(d) glycoproteins: vitronectin (VTN),
(e) other groups: alpha-2-macroglobulin (A2M), clusterin (CLU), and kininogen-1 (KNG1).

However, a cluster of eight proteins implicated in the complement and coagulation cascades was only observed in the profile of non-responders: C4b-binding protein alpha chain (C4BPA), complement C5 (C5), complement factor I (CFI), complement factor H (CFH), complement factor H-related protein 1 (CFHR1), alpha-1-antitrypsin (SERPINA1), prothrombin (F2), and plasminogen (PLG) (see Figure 2 and Table 3). 

The second most abundant group of proteins identified in the serum samples of HER2-positive BC before NAC is formed by apolipoproteins (see Figure 2). From the seven different apolipoproteins identified in the sera of HER2-positive BC patients, five proteins were commonly identified in both responders and non-responders: apolipoprotein A-I (APOA1), apolipoprotein A-IV (APOA4), apolipoprotein B-100 (APOB), apolipoprotein E (APOE), and apolipoprotein M (APOM). While apolipoprotein C-III (APOC3) was only found in the serum of responders, apolipoprotein D (APOD) was identified in the sera of non-responders.

Aside from APOD and the eight proteins involved in complement and coagulation cascade pathways, five unique proteins were identified in the serum of non-responders: immunoglobulin lambda-like polypeptide 5 (IGLL5), CD5 antigen-like (CD5L), afamin (AFM), ficolin-3 (FCN3), and haptoglobin-related protein (HPR). In the case of responders, aside from APOC3, two unique proteins were also identified: gelsolin (GSN) and immunoglobulin kappa constant (IGKC).

Furthermore, the analysis by STRING also revealed that a total of eight proteins implicated in platelet degranulation (ALB, AHSG, APOH, FN1, HRG, ITIH3, ITIH4, TF) and seven proteins that participate in the regulation of immune system processes (PGLYRP2, CD5L, CPN2, GSN, HPX, AMBP, FCN3, RBP4) were commonly identified in the serum of both groups, responders and non-responders (see Figure 2).

### 3.3. Differential Protein Expression

Serum samples separated by sodium dodecyl sulfate-polyacrylamide gel electrophoresis (SDS-PAGE) and processed following **method 1** described in Section 2.7 were then quantitatively analyzed by the emerging proteomic platform for label-free quantification SWATH-MS.

The comparison of the protein patterns allowed the identification of differentially expressed proteins between responders and non-responders. Results were filtered to present a *p*-value ≤ 0.05 and interestingly, *n* = 38 proteins were found to be differentially expressed, of which *n* = 26 were upregulated and *n* = 12 downregulated in responders (see Table 4). 

**Figure 3:**
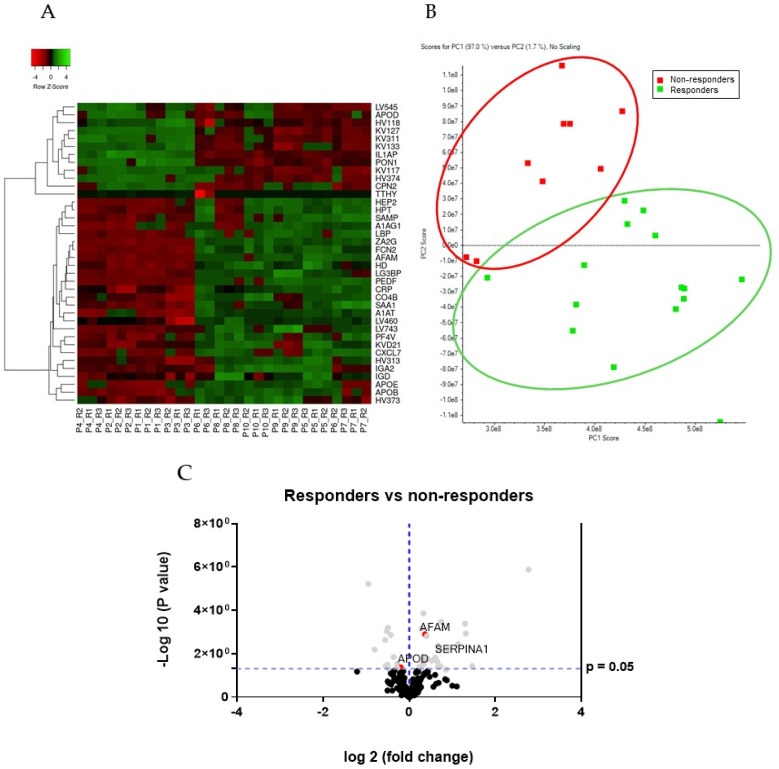
Unsupervised analysis of differentially expressed proteins in serum from responders vs. non-responders to NAC by SWATH-MS. (**A**) Heat map showing hierarchical clustering between responders and non-responders to NAC using the top 38 differentially expressed proteins. Protein expression values were z-score normalized prior to clustering. (**B**) PCA analysis showing the separation of samples from responders (green) and non-responders (red) to NAC. (**C**) Volcano diagram resulting from the statistical analysis of the 306 proteins (library proteins) quantified among responder and non-responder groups. Proteins are separated according to the log2 of the FCh (*x*-axis) and the −log10 of the *p*-values based on a two-tailed t-test (*y*-axis).

The unsupervised hierarchical clustering analysis (heat map) demonstrated clear discrimination between the two groups of samples (responders and non-responders) (see Figure 3A). Furthermore, PCA, which is another unsupervised method, clearly revealed that the samples of the responders and non-responders’ patients were separated in the PC1 axis, which explains 97.0% of the variance between the samples (see Figure 3B). Volcano plots of the global quantification of proteins between responder and non-responder patients were generated by plotting the log 2-fold changes for the identified proteins against their corresponding adjusted *p*-value (see Figure 3C).

To interpret global changes in the serum proteome associated with response/resistance to NAC, the 38 proteins whose levels were significantly different between responder and non-responder patients following method 1 were analyzed using the STRING software. The analysis revealed that the acute-phase response pathway was mainly associated with 7 of 38 dysregulated serum proteins. Particularly, the cluster of acute-phase response proteins found to be downregulated in non-responders is formed by: CRP (C-reactive protein), SAA1 (Serum amyloid A-1 protein), HP (Haptoglobin), APCS (Serum amyloid P-component), SERPINA1 (Alpha-1-antitrypsin), LBP (Lipopolysaccharide-binding protein), ORM1 (Alpha-1-acid glycoprotein 1) (see Figure S3). Furthermore, aside from CRP, SERPINA1 and APCS, two proteins implicated in the complement activation were also found to be downregulated in the non-responders group: FCN2 and C4B.

### 3.4. Comparison of the Serum Proteomic Profile Common to the Three Methods Obtained by Shotgun (DDA Analysis) and SWATH-MS in HER2-Positive BC Patients before NAC

**Figure 4:**
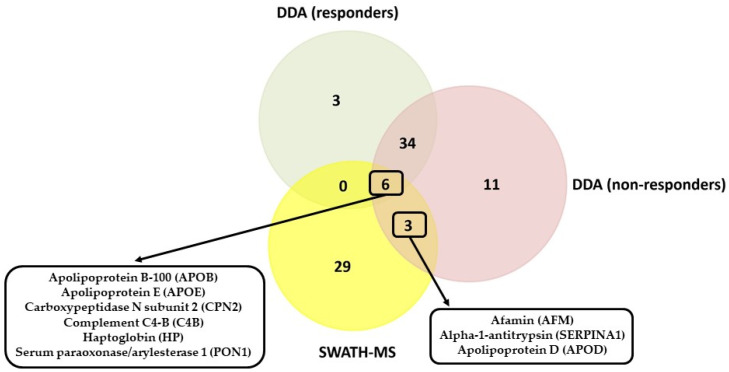
Comparison of the results obtained by the DDA analysis (qualitative) and the SWATH-MS analysis (quantitative).

The 43 proteins identified in responders and 54 proteins identified in non-responders common to the three different methods of sample treatment were compared with the results obtained by SWATH-MS (see Figure 4). These results confirm that six proteins were presented in the sera of both groups, responders and non-responders: apolipoprotein B-100 (APOB), apolipoprotein E (APOE), carboxypeptidase N subunit 2 (CPN2), complement C4-B (C4B), haptoglobin (HP), and serum paraoxonase/arylesterase 1 (PON1). However, while PON1 and CPN2 were found to be upregulated in non-responders (or downregulated in responders), APOE, HP, C4B, and APOB were found to be downregulated in non-responders (or upregulated in responders). 

This quantitative analysis confirms that three proteins were presented in the sera of non-responders: afamin (AFM), alpha-1-antitrypsin (SERPINA1), and apolipoprotein D (APOD). 

**Figure 5:**
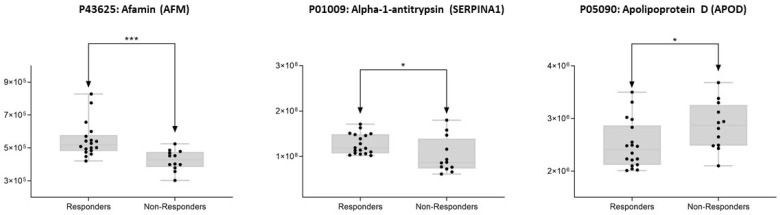
Box plots depicting the three-serum protein AFM, SERPINA1, and APOD levels in each of the study groups (responders and non-responders to NAC). Each data point represents the median value from a single sample. The line inside the box represents the median of all obtained values. The box’s upper and lower limits represent the first and third quartiles, respectively. Whiskers represent the lowest and highest values within 1.5 times the interquartile range. Outliers are any data points that are not included between the whiskers. * *p* < 0.05; *** *p* < 0.001.

Figure 5 shows the mean values of the area obtained for each sample in each group (responders and non-responders) for AFM, SERPINA1, and APOD proteins. These proteins presented the following individual AUC values: AFM with 0.96 (95% CI, 0.842–1), SERPINA1 with 0.62 (95% CI, 0.143–1), and APOD with 0.54 (95% CI, 0.092–0.990). From them, AFM allowed better accurate discrimination between responders and non-responders with a sensitivity of 83.3% and specificity of 100%. These validated proteins play an important biological function in BC and will provide a new target for the effective diagnosis and treatment of BC [67,68,69], and particularly to predict NAC requirements in patients with BC [70]. However, further studies are needed to determine whether this marker can be used as an adjunct test to predict NAC requirements in patients with BC.

### 3.5. In Silico Validation Analysis

To validate the results obtained in the present work, we will compare the quantitative proteomic data obtained by SWATH-MS in HER2-positive BC patients before NAC with the recent previously reported data by Ting Yang et al. [45]. These authors performed an isobaric Tandem Mass Tag (TMT) label-based quantitative proteomic analysis of six serum samples from primary HER2-positive breast cancer cases, including three trastuzumab-based therapy-resistant and three trastuzumab-based therapy responsive cases, to discover the serum fingerprint proteins that are related to the trastuzumab-based therapy response in a parallel manner. Statistically significant differences between the trastuzumab-based therapy-resistant and control trastuzumab-based therapy responsive serum samples (*p* < 0.05 and a fold change ≥ 1.5) showed that 13 secreted serum proteins were upregulated and 5 secreted serum proteins were downregulated. The MS-based proteomics data have been deposited to the ProteomeXchange Consortium and are available via ProteomeXchange with identifier PXD016655. These data are collected in the Supplemental Material (Table S6).

**Figure 6:**
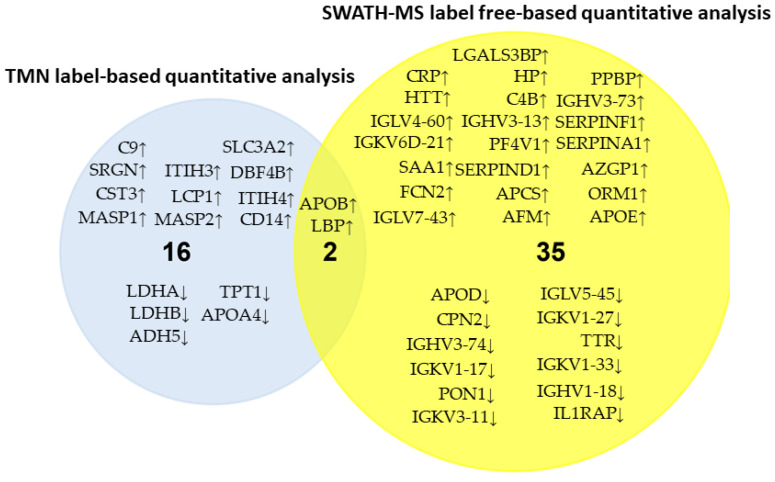
In silico validation after comparing the serum samples analysis from primary HER2-positive breast cancer cases to discover circulating proteins related to the NAC response with two quantitative proteomic methods: the isobaric TMT label-based and the SWATH-MS label-free (↓ denoted downregulation, ↑ denoted upregulation).

The 38 differentially expressed proteins (26 upregulated and 12 downregulated in responders) found by our group were compared with the 18 secreted serum proteins (13 upregulated and 5 downregulated in responders) identified by Ting Yang et al. [45] (see Figure 6). Two proteins were identified by both research groups APOB (apolipoprotein B-100) and LBP (lipopolysaccharide-binding protein). Importantly, these “in silico” comparisons confirm the upregulation of both proteins in the responders patients.

## 4. Discussion

There is an urgent need to identify HER2 positive BC patients who may respond to NAC and to select resistant patients for optional anti-HER2 regents. In the current work, we developed an exhaustive qualitative and quantitative proteomics analysis to investigate differences in the circulating proteins levels between responders and non-responders to NAC. 

Among the two cohorts of HER2-positive patients receiving NAC, we discovered that proteins implicated in the complement and coagulation pathways constitute a signature that is significantly related to the NAC effect. Particularly, a cluster of 8 proteins implicated in the complement and coagulation cascades was only observed in the profile of non-responders: C4b-binding protein alpha chain (C4BPA), complement C5 (C5), complement factor I (CFI), complement factor H (CFH), complement factor H-related protein 1 (CFHR1), alpha-1-antitrypsin (SERPINA1), prothrombin (F2), and plasminogen (PLG). 

Blood coagulation proteins play an important role in tumor growth, according to several studies [50,52,71]. These studies looked at the effects of blood clotting cascade activation on primary tumor growth [72], tumor metastasis, and cancer-associated thrombosis [73], as well as anticancer treatments that target blood-coagulation-associated proteins [74]. Particularly, SERPINA1, a serine protease inhibitor that belongs to the protease inhibitor family, is synthesized and released by tumor cells and is involved in a variety of physiological and pathologic processes including angiogenesis, tumor invasion, and metastasis. [75]. In the case of BC, various studies support blood coagulation proteins as an important patient factor that promotes metastatic potential [76]. For example, when compared to early BC patients, metastatic patients had significantly higher D-dimer values [77]. Furthermore, high plasma fibrinogen levels were linked to a poor response to trastuzumab treatment in HER2 positive BC patients [40] and circulating levels of factor VIII (FVIII) were found to be significantly related to axillary lymph node involvement, the number of metastatic nodes, and HER2 status [78]. These studies, which are consistent with the current work, suggest that measuring some coagulation-related biomarkers could provide additional data for assessing the prognosis of HER2-positive BC patients and could be novel molecular targets.

As mentioned above, the emerging functions of the pro- and anti-coagulant pathways in cell signaling and regulation of extracellular microenvironments give new perspectives on challenges and opportunities in treating cancer patients with anticoagulants [74]. In the pioneering studies by Leo Zacharski [79], anticoagulation by blocking the activity of Gla-domain-containing proteins with Vitamin K antagonists produced a remarkable survival benefit in patients with small-cell lung cancer. It will be of interest for future studies to better understand how cancer cells utilize the coagulant and anticoagulant pathways in the tumor microenvironment (TME) and metastatic niches for survival and the escape from cytotoxic cancer therapy. 

Similar to the blood coagulation proteins, complement proteins, through their interactions with cells in the tumor microenvironment and metastasis-targeted organs, modulate tumor growth, anti-tumor immunity, angiogenesis, and thus overall malignancy progression and, possibly, cancer susceptibility to various therapies [80]. 

In this sense, previous proteomic investigations reported elevated amounts of several complement system components in the sera of colorectal cancer patients [81,82], bladder cancer [52], and in serum and plasma of patients with BC [50,83]. Particularly, a panel of five serum proteins, including complement factor C3a was able to predict the 5-year metastasis-free survival in BC patients [84]. Michlmayr et al. [85] highlighted the role of complement as an important host response factor that could be used to identify early BC patients that are non-responders to NAC. In terms of complement’s immunostimulatory vs. immunoregulatory functions and their potential applications in the development of novel therapies for cancer patients, the only complement inhibitors approved are those that act at the C5 level, such as Eculizumab [86]. It allows complement activation while preventing the formation of the C5a anaphylatoxin and the membrane attack complex. Furthermore, similar to the current study, it was demonstrated that a low level of complement activation is required for an effective response to treatment such as chemotherapy and radiotherapy [87,88].

Apolipoproteins in the blood transfer lipids to cancer cells, providing energy for cancer cell proliferation and invasion, and they also play important roles in cellular signal transduction. A growing body of evidence suggests that apolipoproteins are linked to numerous types of carcinogenesis, such as BC [69,89,90].

In the present work, we discovered that apolipoproteins profile also constitutes a signature that is significantly related to the NAC effect. Particularly, while APOC3 was only observed in the profile of responders, APOD was observed in the profile of non-responders (see Figure 2). Different studies also support the role of apolipoproteins as predictors of treatment response. For example, the apolipoprotein Ea4 allele predicted a better response to donepezil therapy in Alzheimer’s disease [91]. Other studies also correlated levels of some serum lipids, such as Apolipoprotein A-I, with neoadjuvant chemoradiotherapy (NACRT) response in advanced rectal cancer [92,93].

Apart from its well-known role in triglyceride metabolism and insulin resistance, new data reveals that APOC3 is connected to various cancers [94,95]. In a study developed by Jian Shi et al. [96], small cell lung cancer (SCLC) patients undergoing NAC before surgery showed significantly increased expression of APOC3, showing that APOC3 may be used to monitor the efficacy of chemotherapy. Molecular evidence suggests that the human APOC3 promoter is activated synergistically by hepatocyte nuclear factor 4, Mdm2, and Smad proteins [97,98]. Mdm2 antagonizes the indirect inhibition of p53 and SHP on APOC3, which is probably the underlying mechanism for the involvement of APOC3 in tumorigenesis and cancer progression, and therefore, its implication in NAC response shown in the present work. 

As it was mentioned above, APOD was only observed in the profile of non-responders. Among the apolipoproteins, APOD was the first to be demonstrated to play a significant role in BC [99,100]. The interactions of APOD with multiple key pathways may be responsible for its effects in BC, including the estrogen receptor (ER), mitogen-activated protein kinase (MAPK), progesterone receptor (PR), cyclo-oxygenase-2 (COX-2), and 5-lipoxygenase (5-LO) pathways [101,102,103,104,105]. APOD is involved in these signaling pathways, and such pathways interact with each other. Particularly, APOD in plasma was found to function as a predictor during tamoxifen treatment in BC [100,106,107,108]. Molecular studies have shown an inhibitory effect of the estrogen-receptor (ER) on ApoD, with up-regulation following tamoxifen treatment, most likely due to ER activity blockage [109]. As a result, combined ER and ApoD positivity may indicate a malfunctioning hormone receptor pathway, resulting in ineffective tamoxifen treatment and an increased risk of relapse [105,109,110,111,112]. In the present work, further studies are necessary to know the molecular pathways affected by the interaction with APOD, responsible for the NAC effect in non-responders.

Aside from APOC3, GSN and IGKC were also found to be related to the NAC effect in responders. GSN, one of the most potent members of the actin-severing superfamily, regulates actin filament assembly and disassembly [113,114]. GSN is involved in many cellular properties that contribute to carcinogenesis phenotypes, including epithelial to mesenchymal transition (EMT), motility, apoptosis, proliferation, and differentiation [115]. Furthermore, GSN appears to play a variety of roles in tumor biology, with evidence pointing to its involvement in tumor suppression and malignant progression [116,117].

GSN expression is regulated differently in various tumors [118,119,120,121,122,123,124]. During the progression of carcinogenesis, biphasic expression of GSN was found in oral cancers [120,121]. GSN expression is reduced in many transformed and malignant cancer cells, including BC [122,123,124]. Evidence indicated that GSN gene loss is one of the most common disorders in invasive and metastatic BC [125,126]. According to research, the GSN protein was found to be deficient in 71% of human sporadic, invasive breast carcinomas and 56% of ductal carcinomas in situ [125,126]. GSN expression may also be associated with survival from malignant BC, and the frequency of GSN deficiency increases significantly with progression to invasive phenotypic cancer cells, according to clinical evidence [125]. Recent studies have found increased GSN expressions in chemoresistant head-and-neck (HNC) [127] and gynecological cancers [128]. These studies suggested that GSN might play important roles in cancer chemoresistance. However, an opposite effect was observed in the present work. 

Concerning IGKC, a previous study found that this protein is mainly expressed in plasma cells as a prognostic marker in node-negative BC [129]. Across all molecular subtypes, higher IGKC expression was associated with longer metastasis-free survival (MFS) [129]. This effect was especially noticeable in patients with estrogen receptor (ER)-negative, highly proliferating BC. Furthermore, IGKC expression was shown to be a predictor of response to anthracycline-based NAC [129,130,131]. Further immunohistochemical studies could confirm that the presence of IGKC-producing tumor-infiltrating plasma cells was associated with a favorable prognosis in patients with node-negative BC patients who did not receive any systemic adjuvant treatment [130]. A significant interaction between the prognostic effect of IGKC in BC patients and tamoxifen was demonstrated for the first time in the adjuvant setting [132].

In summary, the stromal immunoglobulin kappa chain (IGKC) has been validated as an immunologic biomarker of prognosis and response to therapy in human BC and other cancers. This validation highlights the critical role of humoral immunity in cancer progression control and has important implications for determining cancer prognosis [133].

In the present work, aside from APOD and the eight proteins implicated in the complement and coagulation pathways, six different proteins were also found to be related to the NAC effect in non-responders: immunoglobulin lambda-like polypeptide 5 (IGLL5), CD5 antigen-like (CD5L), afamin (AFM), ficolin-3 (FCN3), haptoglobin-related protein (HPR). The role of these proteins in oncogenesis is not fully understood. Only one study supports the use of CD5L as a therapy to specifically target and destroy cancer cells via complement activation. [134]. Although AFM expression showed no significant prognostic value in BC [135], a significant association between AFM plasma concentrations and clinical outcomes (response to therapy and survival rates) was observed in ovarian cancer [136]. FCN3, a circulating pattern recognition molecule of the lectin pathway, plays a role in host immune responses to cancer [137]. 

In a recent study [138], the potential of FCN3 in the therapeutic intervention of human leiomyoma was demonstrated. Finally, HPR levels in the serum of some cancer patients were found to be elevated with tumor progression, but the relevance of this observation is not understood [139].

The qualitative analysis confirmed that three proteins (AFM, SERPINA1, APOD) were correlated with NAC resistance because they were identified and quantified in the sera of non-responders. The increase in APOD expression and the decrease in AFM and SERPINA1 expression is thus a signature that is significantly related to trastuzumab-based therapeutic resistance. Furthermore, the up-regulation of APOB and LBP in the responder group was supported by the serum proteomic analysis of trastuzumab-based therapy resistant patients before therapy, whose results were reported by T. Yang et al. [45]. This investigation highlights the potential use of serum protein signatures to predict the therapeutic efficacy of NAC in the clinic.

## 5. Conclusions

The results of this study suggest that the identification of some complement and coagulation related circulating proteins constitute a signature that is significantly related to the NAC effect in HER2+ BC patients. Particularly, a low level of complement and coagulation activation is needed to have an effective response to NAC. Circulating apolipoproteins also developed an important role in NAC response being APOC3 and APOD associated with the response and resistance to the treatment, respectively. The increase in APOD expression and the decrease in AFM and SERPINA1 expression correspondingly constitute a signature that is significantly related to the NAC resistance. The upregulation of APOB and LBP in the responder group was also confirmed by an in silico analysis. The results of this study could represent a useful tool to support clinical decision-making in HER2-positive BC patients, providing additional data for the evaluation of NAC response and as potential molecular targets. However, further studies are necessary to know the molecular pathways affected by the interaction with these circulating proteins responsible for the NAC effect in responders and non-responders.

## Figures and Tables

**Table 1:** Clinical characteristics of the patient study group.

Pat. No.	Age	Type	Tumor Size	T-Stage	N-Stage	ER	PR	HER-2	Grading	Response Group
1	61	Ductal	3.4	2	−	+	+	A	1	NR
2	39	Ductal	2.6	2	+	+	+	A	1	NR
3	55	Ductal	2.5	2	+	−	−	A	2	NR
4	58	Ductal	2.4	2	−	−	−	A	2	NR
5	43	Ductal	2.4	2	−	+	−	A	2	R
6	36	Ductal	3.5	2	−	+	+	A	2	R
7	62	Ductal	3.2	2	−	+	+	A	3	R
8	64	Ductal	3	2	+	+	−	A	2	R
9	70	Ductal	2.4	2	−	−	−	A	3	R
10	44	Ductal	5.5	3	−	−	−	A	2	R

Abbreviations: ER = estrogen receptor; PR = progesterone receptor, HER-2 = human epidermal growth factor receptor; NR = non-responder; R = responder; A = amplified.

**Table 2:** Venn diagrams and table showing the number of proteins identified in the sera of HER2-positive BC patients (*n* = 6 responders, *n* = 4 non-responders) obtained before NAC by each treatment method and common to the three methods (**method 1:** analysis of the crude serum; **method 2:** AuNPs-PC analysis; **method 3:** PtNPs-PC analysis).

	Fraction	Number of Proteins Identified			
		Total			Common
Classification		Without NPs (method 1)	With AuNPs (method 2)	With PtNPs (method 3)	
Responders (*n* = 6)		129	61	56	43
Non-responders (*n* = 4)		138	100	61	54
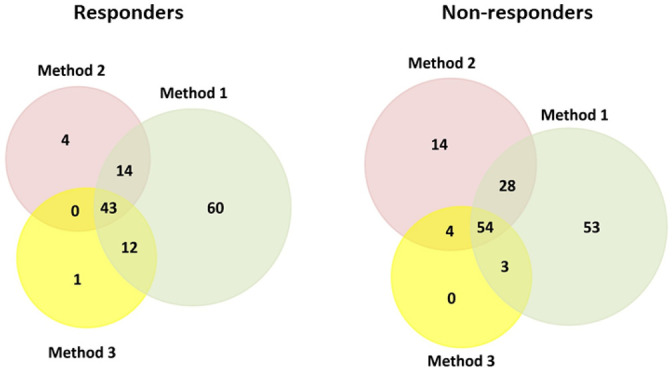					

**Table 3:** Proteins commonly identified by the three different treatment methods of serum samples obtained from HER2-positive BC patients before NAC (*n* = 6 responders, *n* = 4 non-responders) (**method 1:** analysis of the crude serum; **method 2:** AuNPs-PC analysis; **method 3:** PtNPs-PC analysis). The accession number, gene name, and species (Human) were reported.

Protein Name	UniProt Name	Entry Name	Gene	Responders	Non-Responders
Apolipoprotein C-III	P02656	APOC3_HUMAN	APOC3	X	
Gelsolin	P06396	GELS_HUMAN	GSN	X	
Immunoglobulin kappa constant	P01834	IGKC_HUMAN	IGKC	X	
Immunoglobulin lambda-like polypeptide 5	B9A064	IGLL5_HUMAN	IGLL5		X
CD5 antigen-like	O43866	CD5L_HUMAN	CD5L		X
Afamin	P43652	AFAM_HUMAN	AFM		X
Plasminogen	P00747	PLMN_HUMAN	PLG		X
Ficolin-3	O75636	FCN3_HUMAN	FCN3		X
Complement factor H	P08603	CFAH_HUMAN	CFH		X
Complement factor H-related protein 1	Q03591	FHR1_HUMAN	CFHR1		X
Alpha-1-antitrypsin	P01009	A1AT_HUMAN	SERPINA1		X
C4b-binding protein alpha chain	P04003	C4BPA_HUMAN	C4BPA		X
Complement factor I	P05156	CFAI_HUMAN	CFI		X
Complement C5	P01031	CO5_HUMAN	C5		X
Apolipoprotein D	P05090	APOD_HUMAN	APOD		X
Haptoglobin-related protein	P00739	HPTR_HUMAN	HPR		X
Prothrombin	P00734	THRB_HUMAN	F2		X
Serum paraoxonase/arylesterase 1	P27169	PON1_HUMAN	PON1	X	X
Immunoglobulin heavy constant gamma 1	P01857	IGHG1_HUMAN	IGHG1	X	X
Inter-alpha-trypsin inhibitor heavy chain H3	Q06033	ITIH3_HUMAN	ITIH3	X	X
Kininogen-1	P01042	KNG1_HUMAN	KNG1	X	X
Plasma protease C1 inhibitor	P05155	IC1_HUMAN	SERPING1	X	X
Inter-alpha-trypsin inhibitor heavy chain H2	P19823	ITIH2_HUMAN	ITIH2	X	X
Vitronectin	P04004	VTNC_HUMAN	VTN	X	X
Vitamin D-binding protein	P02774	VTDB_HUMAN	GC	X	X
Inter-alpha-trypsin inhibitor heavy chain H1	P19827	ITIH1_HUMAN	ITIH1	X	X
Complement C1q subcomponent subunit C	P02747	C1QC_HUMAN	C1QC	X	X
Antithrombin-III	P01008	ANT3_HUMAN	SERPINC1	X	X
Fibronectin	P02751	FINC_HUMAN	FN1	X	X
Apolipoprotein A-I	P02647	APOA1_HUMAN	APOA1	X	X
Complement C2	P06681	CO2_HUMAN	C2	X	X
Hemopexin	P02790	HEMO_HUMAN	HPX	X	X
Apolipoprotein E	P02649	APOE_HUMAN	APOE	X	X
Immunoglobulin heavy constant alpha 1	P01876	IGHA1_HUMAN	IGHA1	X	X
N-acetylmuramoyl-L-alanine amidase	Q96PD5	PGRP2_HUMAN	PGLYRP2	X	X
Haptoglobin	P00738	HPT_HUMAN	HPT	X	X
Alpha-2-macroglobulin	P01023	A2MG_HUMAN	A2M	X	X
Vitamin K-dependent protein S	P07225	PROS_HUMAN	PROS1	X	X
Immunoglobulin heavy constant mu	P01871	IGHM_HUMAN	IGHM	X	X
Serotransferrin	P02787	TRFE_HUMAN	TF	X	X
Clusterin	P10909	CLUS_HUMAN	CLU	X	X
Alpha-2-antiplasmin	P08697	A2AP_HUMAN	SERPINF2	X	X
Carboxypeptidase N subunit 2	P22792	CPN2_HUMAN	CPN2	X	X
Albumin	P02768	ALBU_HUMAN	ALB	X	X
Complement factor B	P00751	CFAB_HUMAN	CFB	X	X
Inter-alpha-trypsin inhibitor heavy chain H4	Q14624	ITIH4_HUMAN	ITIH4	X	X
Retinol-binding protein 4	P02753	RET4_HUMAN	RBP4	X	X
Complement C1q subcomponent subunit B	P02746	C1QB_HUMAN	C1QB	X	X
Complement C4-B	P0C0L5	CO4B_HUMAN	C4B	X	X
Apolipoprotein A-IV	P06727	APOA4_HUMAN	APOA4	X	X
Alpha-2-HS-glycoprotein	P02765	FETUA_HUMAN	AHSG	X	X
Beta-2-glycoprotein 1	P02749	APOH_HUMAN	APOH	X	X
Complement C3	P01024	CO3_HUMAN	C3	X	X
Apolipoprotein M	O95445	APOM_HUMAN	APOM	X	X
Protein AMBP	P02760	AMBP_HUMAN	AMBP	X	X
Apolipoprotein B-100	P04114	APOB_HUMAN	APOB	X	X
Histidine-rich glycoprotein	P04196	HRG_HUMAN	HRG	X	X

**Table 4:** Specific differentially expressed proteins detected in non-responder patients relative to the responders’ group after the analysis of serum samples (**method 1**) by SWATH-MS. The fold change ratio (FCh) was calculated as the ratio of the geometric mean of the samples, corresponding to the calculation of the normal arithmetic ratio of the logarithmic transformation and inverse transformation regions (↓ denoted downregulation, ↑ denoted upregulation).

Uniprot Code	Gene Name	Protein Name	*p*-Value	FCh	Response to NAC
P02741	CRP	C-reactive protein	0.00000134	6.829624202	↓Non-responders
P0DOX3	N/A	Immunoglobulin delta heavy chain	0.036959856	2.75912755	↓Non-responders
P42858	HTT	Huntingtin	0.001165915	2.485533233	↓Non-responders
A0A075B6I1	IGLV4-60	Immunoglobulin lambda variable 4-60	0.000406597	2.458347205	↓Non-responders
A0A0A0MT36	IGKV6D-21	Immunoglobulin kappa variable 6D-21	0.003581497	2.197533513	↓Non-responders
P0DJI8	SAA1	Serum amyloid A-1 protein	0.00604557	1.859220088	↓Non-responders
Q15485	FCN2	Ficolin-2	0.000332323	1.677931316	↓Non-responders
P04211	IGLV7-43	Immunoglobulin lambda variable 7-43	0.037948547	1.658779213	↓Non-responders
Q08380	LGALS3BP	Galectin-3-binding protein	0.006599706	1.630292329	↓Non-responders
P00738	HP	Haptoglobin	0.004228108	1.588362659	↓Non-responders
A0A0B4J1V6	IGHV3-73	Immunoglobulin heavy variable 3-73	0.040907506	1.586899833	↓Non-responders
P0DOX2	N/A	Immunoglobulin alpha-2 heavy chain	0.023074607	1.573627149	↓Non-responders
P0C0L5	C4B	Complement C4-B	0.015214866	1.524647235	↓Non-responders
P01766	IGHV3-13	Immunoglobulin heavy variable 3-13	0.021240441	1.437890543	↓Non-responders
P10720	PF4V1	Platelet factor 4 variant	0.016361485	1.340407061	↓Non-responders
P05546	SERPIND1	Heparin cofactor 2	0.001552533	1.328820011	↓Non-responders
P02743	APCS	Serum amyloid P-component	0.019065612	1.322698566	↓Non-responders
P43652	AFM	Afamin	0.001267906	1.29052425	↓Non-responders
P02775	PPBP	Platelet basic protein	0.016993573	1.271442298	↓Non-responders
P36955	SERPINF1	Pigment epithelium-derived factor	0.000136647	1.257360069	↓Non-responders
P04114	APOB	Apolipoprotein B-100	0.030902399	1.257016742	↓Non-responders
P01009	SERPINA1	Alpha-1-antitrypsin	0.030398754	1.252498148	↓Non-responders
P18428	LBP	Lipopolysaccharide-binding protein	0.017544605	1.251259211	↓Non-responders
P25311	AZGP1	Zinc-alpha-2-glycoprotein	0.000669343	1.225228845	↓Non-responders
P02763	ORM1	Alpha-1-acid glycoprotein 1	0.041098354	1.176485834	↓Non-responders
P02649	APOE	Apolipoprotein E	0.048165556	1.174660228	↓Non-responders
P05090	APOD	Apolipoprotein D	0.042743468	0.873825169	↑ Non-responders
P22792	CPN2	Carboxypeptidase N subunit 2	0.029951414	0.826212759	↑ Non-responders
A0A0B4J1X5	IGHV3-74	Immunoglobulin heavy variable 3-74	0.043884568	0.824616939	↑ Non-responders
P01599	IGKV1-17	Immunoglobulin kappa variable 1-17	0.014360162	0.779005563	↑ Non-responders
P27169	PON1	Serum paraoxonase/arylesterase 1	0.001360555	0.744636131	↑ Non-responders
P04433	IGKV3-11	Immunoglobulin kappa variable 3-11	0.000634898	0.712186884	↑ Non-responders
A0A087WSX0	IGLV5-45	Immunoglobulin lambda variable 5-45	0.032089451	0.703275957	↑ Non-responders
A0A075B6S5	IGKV1-27	Immunoglobulin kappa variable 1-27	0.00090962	0.698043088	↑ Non-responders
Q5U7I5	TTR	Transthyretin	0.03635507	0.684353438	↑ Non-responders
P01594	IGKV1-33	Immunoglobulin kappa variable 1-33	0.002337947	0.679286204	↑ Non-responders
A0A0C4DH31	IGHV1-18	Immunoglobulin heavy variable 1-18	0.006452433	0.573322357	↑ Non-responders
Q9NPH3	IL1RAP	Interleukin-1 receptor accessory protein	0.00000586	0.518752156	↑ Non-responders

## Data Availability

The data presented in this study are available in this article (and Supplementary Material).

## Supplementary Materials

The following are available online at https://www.mdpi.com/article/10.3390/cancers14041087/s1, Annex 1: Criteria for HER2-targeted NAC in HER2-positive BC patients, Annex 2. Synthesis of inorganic nanoparticles [47], Figure S1: TEM image of AuNPs@citrate in aqueous phase and the characterization data, Figure S2: TEM image of PtNPs@citrate in aqueous phase and the characterization data, Figure S3. Cluster of acute-phase response proteins found in the protein–protein interaction network map of the genes encoded differentially regulated proteins for the responders’ patients found after the proteomic analysis of the serum samples (**method 1**), Table S1: Proteins identified in the crude serum samples (**method 1**) belonging to HER2-positive BC patients that were obtained before starting the neoadjuvant treatment. These patients showed a different response after the NAT treatment: responders (*n* = 6), non-responders (*n* = 4). The accession number, gene name and species (Human) were reported, Table S2: Proteins identified in the serum samples pretreated with AuNPs (**method 2**) belonging to HER2-positive BC patients that were obtained before starting the neoadjuvant treatment. These patients showed a different response after the NAT treatment: responders (*n* = 6), non-responders (*n* = 4). The accession number, gene name and species (Human) were reported, Table S3: Proteins identified in the serum samples pretreated with PtNPs (**method 3**) belonging to HER2-positive BC patients that were obtained before starting the neoadjuvant treatment. These patients showed a different response after the NAT treatment: responders (*n* = 6), non-responders (*n* = 4). The accession number, gene name and species (Human) were reported, Table S4. List of 43 common proteins identified in the serum of responders pretreated by the three different methods (with and without NPs). The accession number, gene name, and species (Human) were reported, Table S5. List of 54 common proteins identified in the serum of non-responders pretreated by the three different methods (with and without NPs). The accession number, gene name, and species (Human) were reported. Table S6. List of the upregulated and downregulated proteins (a fold change ≥ 1.5 and *p* < 0.05) found after the TMT labeling-based quantitative proteomic analysis of 6 serum samples from primary HER2-positive breast cancer cases, including 3 trastuzumab-based therapy-resistant and 3 trastuzumab-based therapy responsive cases developed by T. Yang et al. [45].
