# Supplementary material for: Circulating Proteins Associated with Response and Resistance to Neoadjuvant Chemotherapy in HER2-Positive Breast Cancer
Source: Cancers (Basel). 2022 Feb 21;14(4):1087. doi: 10.3390/cancers14041087 (PMC8870308; doi:10.3390/cancers14041087)
Supplement: Supplementary file 1 [file cancers-14-01087-s001.zip › cancers-1548248-supplementary.pdf]

ANNEXES

ANNEXE 1. Criteria for HER2-targeted NAC in HER2-positive BC patients

- Inclusion criteria

1. Women with ages ≥ 18 years with early high-risk or locally advanced BC (T1cN1; T2N1; T2N0; T3N0) (stage III according to AJCC) suitable for neoadjuvant treatment.

2. Histologically confirmed unilateral invasive BC.

3. HER2 positive disease according to the 2013 ASCO/CAP guidelines [defined as IHC 3+ or positive ISH (by gene copy number or HER2/CEP17 gene ratio of 2 or more)].

4. Known estrogen receptor (ER) and progesterone receptor (PgR).

5. The availability of a representative Formalin-Fixed Paraffin-Embedded (FFPE) tumor block taken at the diagnostic biopsy for central confirmation of HER2 eligibility, for evaluation of ER, PgR, Ki67, and for evaluation of biomarkers is mandatory. Note: The diagnostic biopsy of the breast lesion may have been taken prior to the required screening procedures. If a diagnostic sentinel node biopsy is performed, an FFPE block should be available. An FFPE tumor block is also mandatory after the first cycle of therapy. Surgical tissue (residual tumor or tumor bed in the case of CRP and axillary lymph node material) is also mandatory.

6. Informed consent for the mandatory collection of blood samples before starting neoadjuvant treatment, after the first cycle of therapy, at the end of neoadjuvant treatment (before surgery), 6 months after surgery and at the end of all treatments.

7. ECOG performance status 0 or 1

8. For women who are not postmenopausal (≥ 12 months of non-therapy-induced amenorrhea) or surgically sterile (absence of ovaries and / or uterus): agreement to maintain abstinence or to use single or combined contraceptive methods that result in a failure rate of <1% per year during the treatment period and for at least 6 months after the last dose of study drugs. Abstinence is only acceptable if it is consistent with the patient's preferred and usual lifestyle. Periodic abstinence (for example, calendar, ovulation, symptothermic, or post-ovulation methods) and abstinence are not acceptable contraceptive methods. Examples of contraceptive methods with a failure rate of <1% per year include tubal ligation, male sterilization, hormonal implants, established and appropriate use of combined oral or injected hormonal contraceptives, and certain intrauterine devices. Alternatively, two methods can be combined (eg, two barrier methods such as a condom and a cervical cap) to achieve a failure rate of <1% per year. Barrier methods should always be supplemented with the use of a spermicide.

9. Written informed consent to participate in the trial (approved by the Independent Ethics Committee [CEIC]) obtained prior to any study-specific selection procedure.

10. Willing and able to comply with protocol.

- Exclusion criteria

1. Evidence of bilateral BC or metastatic disease (M1).

2. HER2 negative patients defined as 0-1+ by immunohistochemistry or 2+ by immunohistochemistry without HER2 amplification by in situ hybridization (ISH) or other amplification tests performed locally are not considered eligible for study.

3. Pregnant or lactating women. Documentation of a negative pregnancy test should be available for premenopausal women with intact reproductive organs and for women less than a year after the last menstrual cycle.

4. Women of childbearing potential, unless (1) they are surgically sterile or (2) use adequate contraceptive measures, for example, abstinence, an intrauterine device, or a double barrier method of contraception.

5. Previous treatment with chemotherapy, hormone therapy, or an investigational drug for any type of malignancy.

6. Investigational pretreatment for any non-malignant condition within 4 weeks of the randomization date.

7. Administration of a live attenuated vaccine within 4 weeks prior to day 1 or anticipation that such live attenuated vaccine will be needed during the study.

8. Previous or concomitant malignancy of any other type that could affect compliance with the protocol or interpretation of results. Patients with curatively treated basal cell carcinoma of the skin or cervical cancer in situ are generally eligible.

9. Pre-existing motor or sensory neuropathy grade > 1 for any reason.

10. History of allergic, anaphylactic, or other hypersensitivity reactions to chimeric or humanized antibodies or fusion proteins.

11. Patients with previous transplantation of allogeneic stem cells or solid organs.

12. Known clinically significant liver disease, including viral, alcoholic, or other active hepatitis, cirrhosis, fatty liver, and inherited liver disease.

13. History of HIV infection, active hepatitis B (chronic or acute), or hepatitis C infection. Patients with past or resolved hepatitis B infection (defined as a negative HBsAg test and a hepatitis B central antigen test [anti-HBc] positive) are eligible. Hepatitis C virus (HCV) antibody positive patients are eligible only if the polymerase chain reaction (PCR) assay is negative for HCV RNA.

14. Active tuberculosis.

15. Serious infections within 4 weeks of day 1, including but not limited to hospitalization for complications of infection, bacteremia, or severe pneumonia. Signs or symptoms of significant infection in the 2 weeks prior to day 1.

16. Received oral or intravenous antibiotics in the 2 weeks prior to Cycle 1 Day 1.

17. Other serious medical illness or condition including: a history of documented congestive heart failure; New York Heart Association (NYHA) CHF Class II or higher; angina pectoris requiring antianginal medication or unstable angina in the 6 months prior to Day 1; evidence of transmural infarction on ECG; myocardial infarction stroke or transient ischemic attack (TIA) in the 6 months prior to day 1; poorly controlled hypertension (eg, systolic> 180 mm Hg or diastolic> 100 mm Hg; however, patients with well-controlled hypertension on medication are eligible); clinically significant valvular heart disease; high-risk uncontrolled arrhythmias.

18. Patients with a history of uncontrolled seizures, central nervous system disorders, or psychiatric disability that the investigator deems clinically important and that prevents informed consent or adversely affects compliance with study drugs.

19. Serious uncontrolled infections (bacterial or viral) or poorly controlled diabetes mellitus.

20. Any of the following abnormal baseline hematology values:

- a. White blood cell count (WBC) <2.5 x 109 / L
- b. Absolute Neutrophil Count (ANC) <1.5 x 109 / L
- c. Lymphocyte count <0.5 x 109 / L
- d. Platelet count <100 x 109 / L
- e. Hemoglobin (Hb) <10 g / dL

21. Any of the following abnormal reference laboratory tests:

- a. Total serum bilirubin> 1.5 x ULN (upper limit of normal) (except for patients with clearly documented Gilbert syndrome)
- b. Alanine transaminase (ALT) or aspartate transaminase (AST)> 1.25 x ULN
- c. to. Alkaline phosphatase> 2.5 x ULN
- d. Serum creatinine> 1.5 x ULN
- e. INR and aPTT> 1.5 × ULN in the 2 weeks prior to enrollment. This applies only to patients who are not receiving therapeutic anticoagulation; patients receiving therapeutic anticoagulation should receive a stable dose.

22. Baseline left ventricular ejection fraction (LVEF) <50% by echocardiography or multigrade scintigraphy (MUGA).

23. Major surgical procedure within 28 days prior to Day 1 or anticipation of the need for a major surgical procedure during the course of the study.

24. Flu vaccination should only be given during flu season (approximately October through March). Patients should not receive the live, attenuated influenza vaccine (e.g. FluMist®) within 4 weeks prior to Day 1 or at any time during the study.

ANNEXE 2. *Synthesis of inorganic nanoparticles*

Synthesis of citrate-gold nanoparticles

AuNPs (10.02 ± 0.91 nm) were synthesized by the citrate reduction method in aqueous solution [47]. Briefly, 60 ml of sodium citrate tribasic solution (0.075% w/v) was heated to 100 °C, and then gold was added as 54 µL of 10% w/v of hydrogen tetrachloroaurate (III) hydrate solution. The reaction mixture was kept under refluxing until a deep red color was detected. Solution of nanoparticles is chilled at room temperature and stored at 4 ºC for a maximum of one month.

and

Synthesis of citrate-capped platinum nanoparticles

PtNPs (2.40 ± 0.30 nm) were synthesized by the method previously described [47]. Glass wares were cleaned in aqua regia and all solutions were prepared using double distilled water. The synthesis of PtNPs was performed in a glass ware with the magnetic stir. Aqueous solutions of H_2PtCl_6 (1 mL, 16 mM) and trisodium citrate (1 mL, 40 mM) were mixed with 38 mL water and stirred for 30 min at room temperature. Subsequently, $NaBH_4$ (200 µL, 50 mM) was added dropwise into the mixture. The colorless reactant mixture immediately turned into brownish yellow. Finally, the mixture was allowed to react and stirred at ambient temperature for 1 h.

**AuNPs@citrate (nm)**			
1	7.94	21	10.35
2	8.37	22	10.38
3	8.38	23	10.41
4	8.60	24	10.44
5	8.78	25	10.56
6	8.78	26	10.65
7	8.87	27	10.65
8	9.17	28	10.68
9	9.19	29	10.80
10	9.41	30	10.81
11	9.49	31	10.83
12	9.49	32	10.85
13	9.55	33	10.86
14	9.62	34	10.86
15	9.82	35	10.88
16	9.84	36	10.95
17	9.89	37	10.96
18	9.89	38	10.99
19	9.99	39	11.48
20	10.03	40	11.50
Count	40		
Mean	10.02		
Minimum	7.94		
Maximum	11.50		
Standar Deviation	0.91		

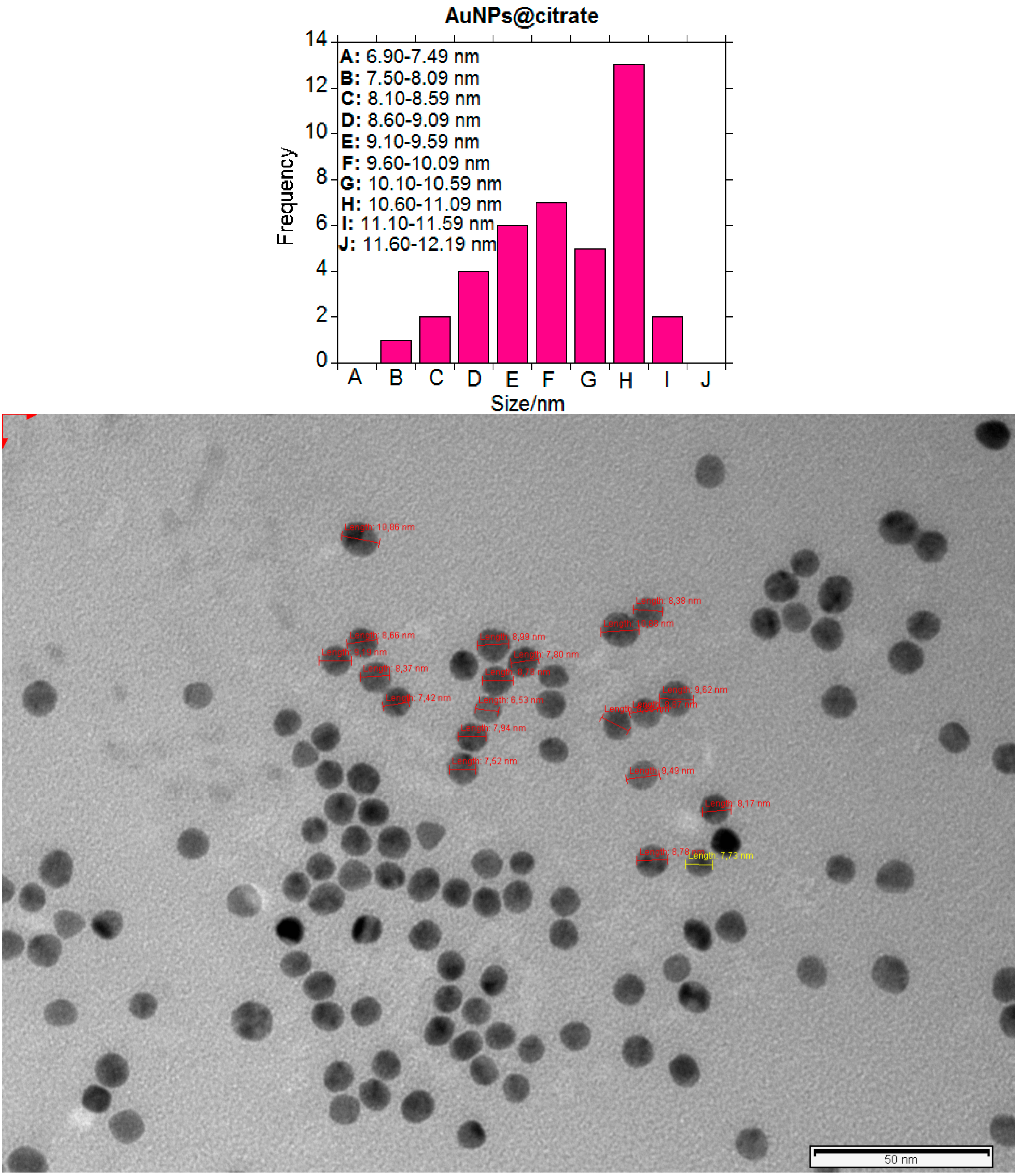

Figure S1. TEM image of AuNPs@citrate in aqueous phase and the characterization data.

PtNPs@citrate (nm)			
1	2.02	21	2.31
2	2.02	22	2.32
3	2.03	23	2.38
4	2.05	24	2.42
5	2.08	25	2.50
6	2.10	26	2.51
7	2.16	27	2.54
8	2.16	28	2.57
9	2.16	29	2.58
10	2.16	30	2.68
11	2.17	31	2.69
12	2.17	32	2.70
13	2.17	33	2.75
14	2.17	34	2.80
15	2.17	35	2.83
16	2.18	36	2.83
17	2.18	37	2.89
18	2.22	38	2.94
19	2.23	39	2.99
20	2.23	40	3.00
Count	40		
Mean	2.40		
Minimum	2.02		
Maximum	3.00		
Standar Deviation	0.30		

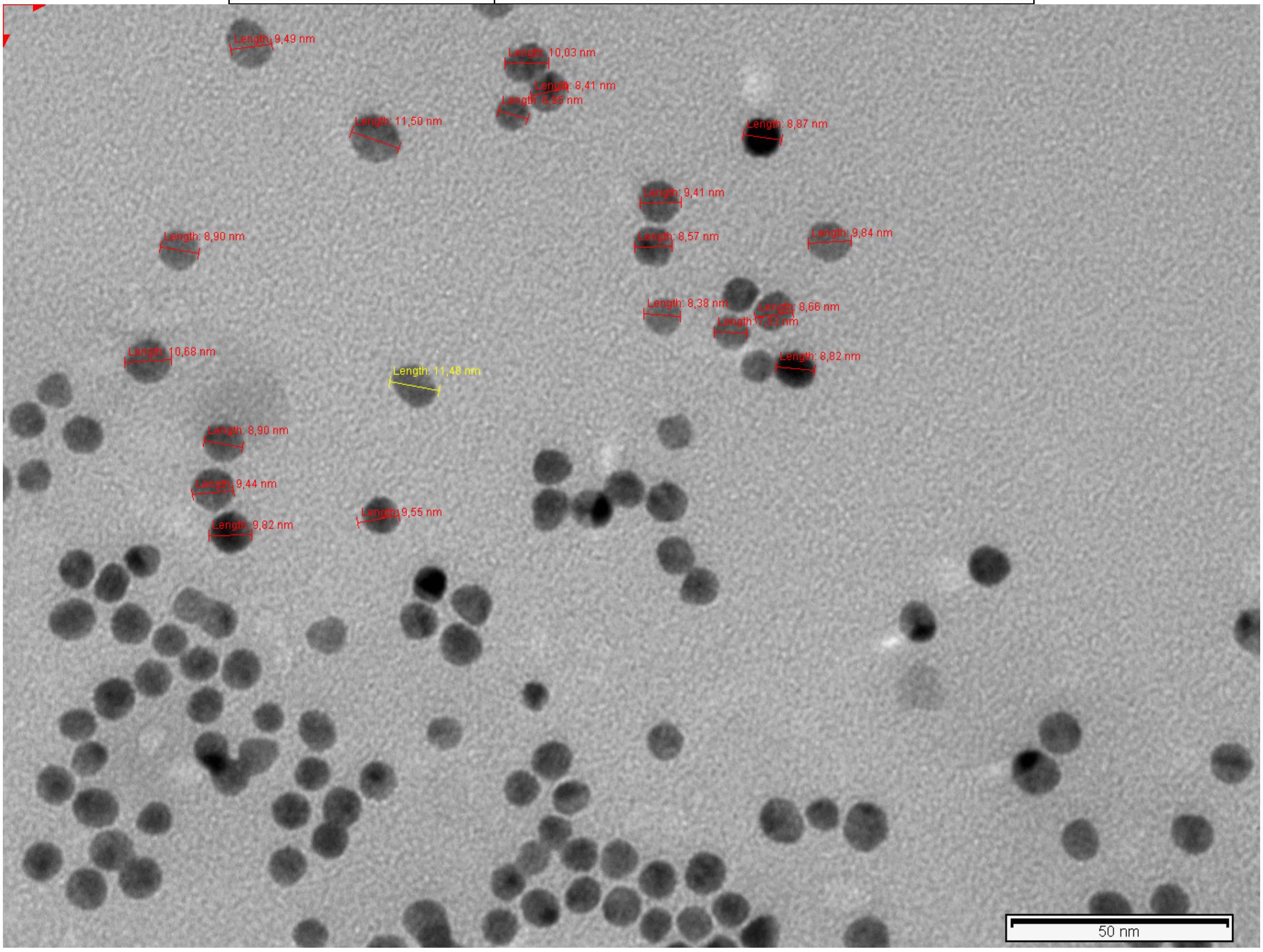

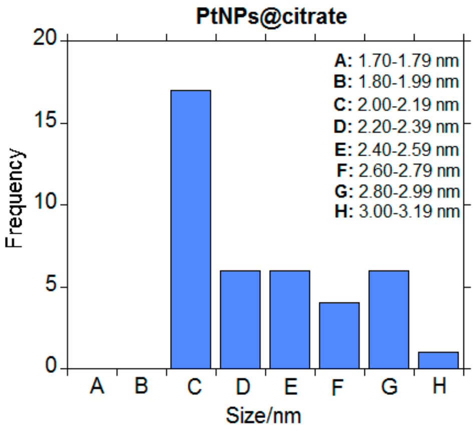

PtNPs@citrate
Frequency
Size/nm
A: 1.70-1.79 nm
B: 1.80-1.99 nm
C: 2.00-2.19 nm
D: 2.20-2.39 nm
E: 2.40-2.59 nm
F: 2.60-2.79 nm
G: 2.80-2.99 nm
H: 3.00-3.19 nm

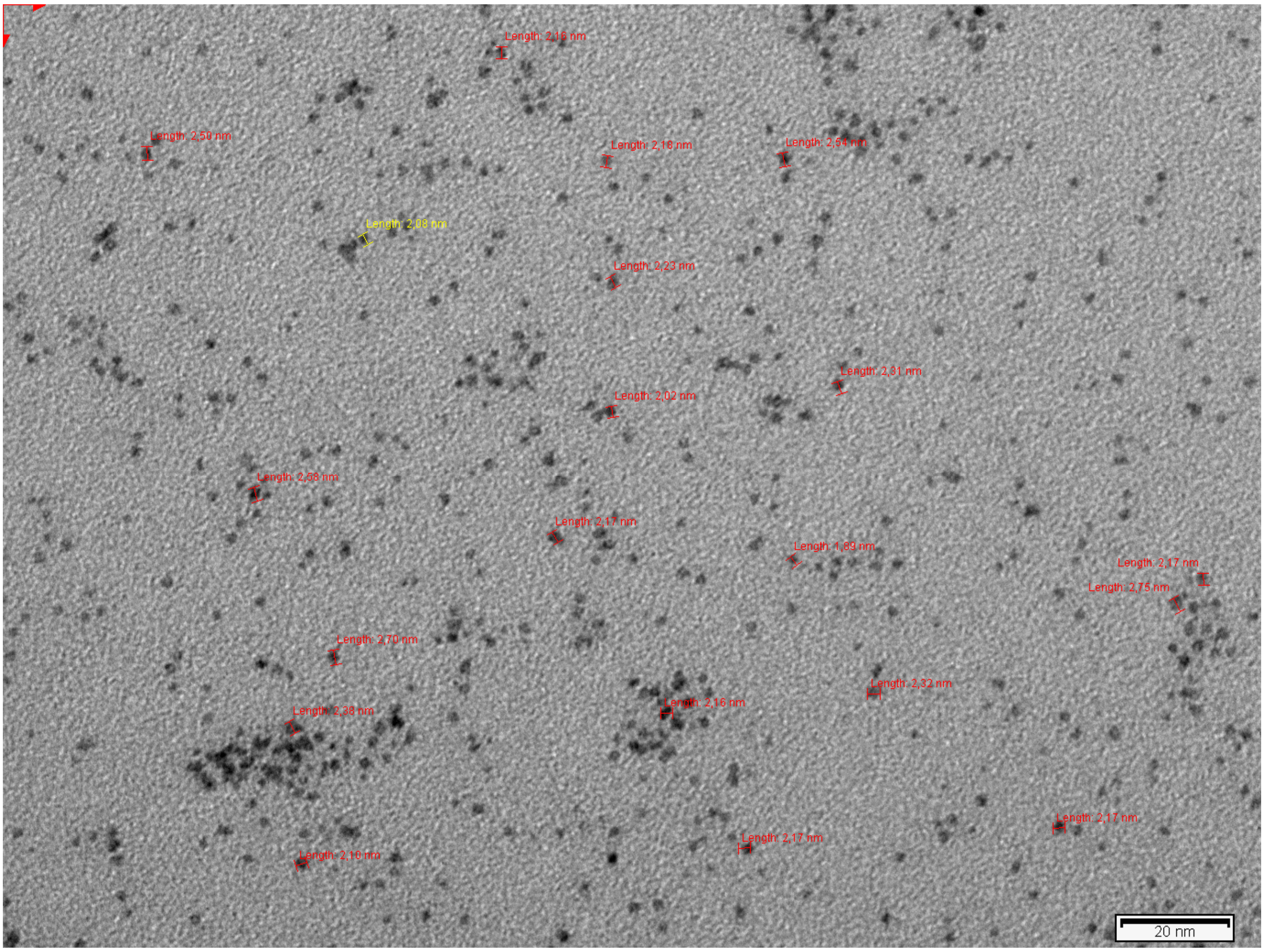

20 nm

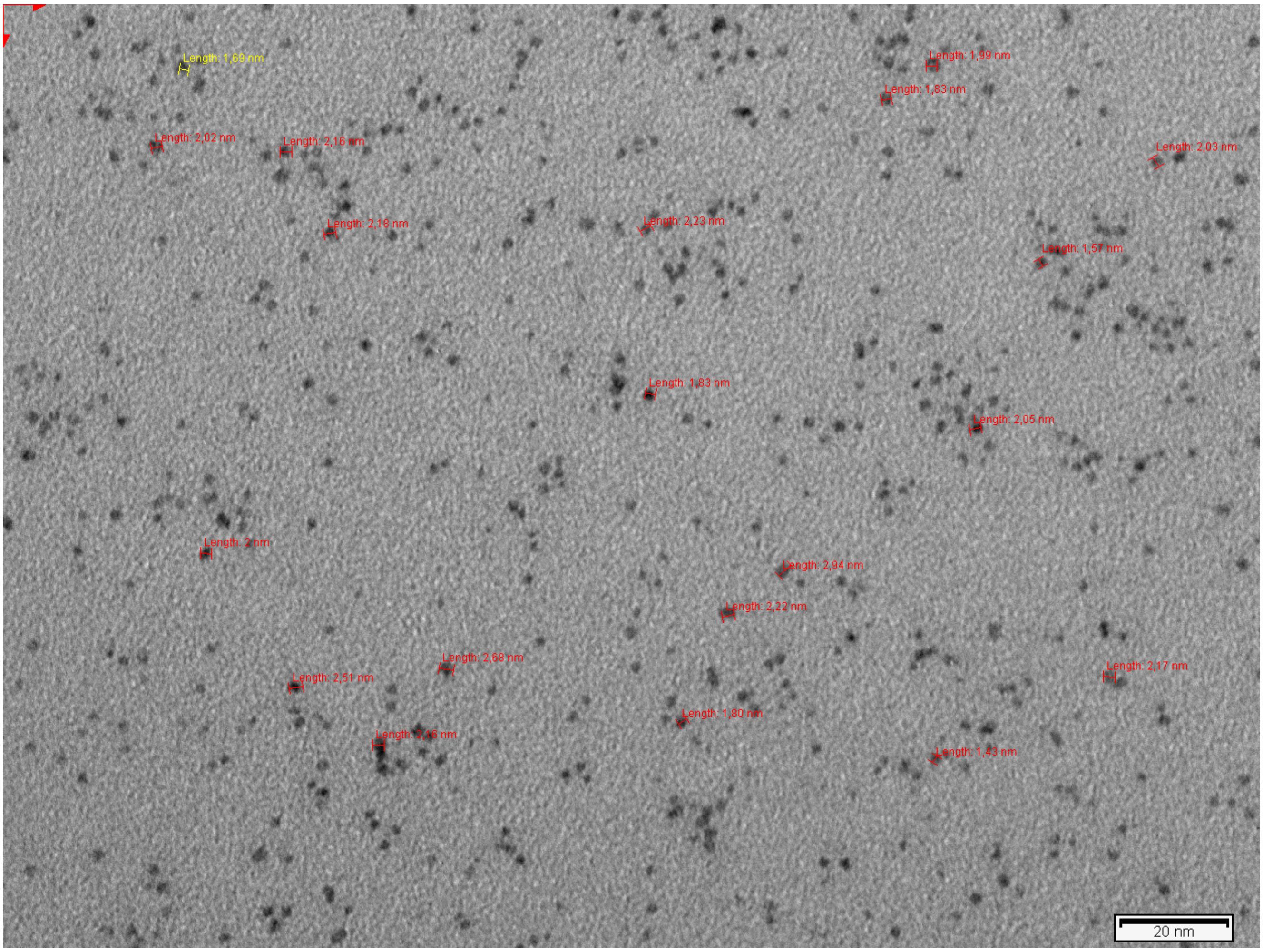

Figure S2. TEM image of PtNPs@citrate in aqueous phase and the characterization data.

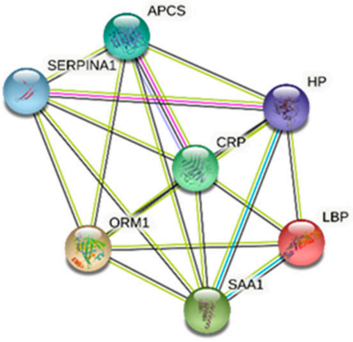

Figure S3. Cluster of acute-phase response proteins found in the protein-protein interaction network map of the genes encoded differentially regulated proteins for the responders patients found after the proteomic analysis of the serum samples (**method 1**).

Table S1. Proteins identified in the crude serum samples **(method 1)** belonging to HER2-positive BC patients that were obtained before starting the neoadjuvant treatment. These patients showed different response after the NAT treatment: responders (*n* = 6), non-responders (*n* = 4). The accession number, gene name and species (Human) were reported.

Protein Name	UniProt Name	Entry Name	Gene	Responders	Non-responders
Complement component C8 gamma chain	P07360	CO8G_HUMAN	C8G	X	
Immunoglobulin lambda variable 9-49	A0A0B4J1Y8	LV949_HUMAN	IGLV9-49	X	
Complement component C8 beta chain	P07358	CO8B_HUMAN	C8B	X	
Prenylcysteine oxidase 1	Q9UHG3	PCYOX_HUMAN	PCYOX1	X	
Fibrinogen alpha chain	P02671	FIBA_HUMAN	FGA	X	
Immunoglobulin lambda variable 3-19	P01714	LV319_HUMAN	IGLV3-19	X	
Serum amyloid A-1 protein	P0DJI8	SAA1_HUMAN	SAA1	X	
Complement component C9	P02748	CO9_HUMAN	C9	X	
Immunoglobulin lambda variable 4-69	A0A075B6H9	LV469_HUMAN	IGLV4-69	X	
Immunoglobulin kappa variable 1D-39	P04432	KVD39_HUMAN	IGKV1D-39	X	
Immunoglobulin kappa variable 1-17	P01599	KV117_HUMAN	IGKV1-17	X	
Pregnancy zone protein	P20742	PZP_HUMAN	PZP		X
Hyaluronan-binding protein 2	Q14520	HABP2_HUMAN	HABP2		X
Immunoglobulin lambda variable 7-43	P04211	LV743_HUMAN	IGLV7-43		X
Immunoglobulin lambda variable 2-11	P01706	LV211_HUMAN	IGLV2-11		X
Immunoglobulin kappa variable 2-30	P06310	KV230_HUMAN	IGKV2-30		X
Properdin	P27918	PROP_HUMAN	CFP		X
Immunoglobulin lambda variable 1-40	P01703	LV140_HUMAN	IGLV1-40		X
Complement C1q subcomponent subunit A	P02745	C1QA_HUMAN	C1QA		X
Coagulation factor X	P00742	FA10_HUMAN	F10		X
Immunoglobulin heavy variable 3-64D	A0A0J9YX35	HV64D_HUMAN	IGHV3-64D		X
Complement factor H-related protein 1	Q03591	FHR1_HUMAN	CFHR1		X
Transferrin receptor protein 1	P02786	TFR1_HUMAN	TFRC		X
Immunoglobulin kappa variable 1-5	P01602	KV105_HUMAN	IGKV1-5		X
Complement factor H-related protein 2	P36980	FHR2_HUMAN	CFHR2		X
Hemoglobin subunit delta	P02042	HBD_HUMAN	HBD		X
Immunoglobulin heavy constant alpha 2	P01877	IGHA2_HUMAN	IGHA2		X
Immunoglobulin lambda variable 8-61	A0A075B6I0	LV861_HUMAN	IGLV8-61		X
Immunoglobulin heavy variable 4-28	A0A0C4DH34	HV428_HUMAN	IGHV4-28		X

Immunoglobulin kappa variable 1-27	A0A075B6S5	KV127_HUMAN	IGKV1-27		X
Beta-2-microglobulin	P61769	B2MG_HUMAN	B2M		X
Apolipoprotein C-III	P02656	APOC3_HUMAN	APOC3	X	X
Serum paraoxonase/ arylesterase 1	P27169	PON1_HUMAN	PON1	X	X
Immunoglobulin heavy constant gamma 1	P01857	IGHG1_HUMAN	IGHG1	X	X
Immunoglobulin heavy variable 5-51	A0A0C4DH38	HV551_HUMAN	IGHV5-51	X	X
Inter-alpha-trypsin inhibitor heavy chain H3	Q06033	ITIH3_HUMAN	ITIH3	X	X
Carboxypeptidase N catalytic chain	P15169	CBPN_HUMAN	CPN1	X	X
Immunoglobulin kappa variable 1-33	P01594	KV133_HUMAN	IGKV1-33	X	X
Kininogen-1	P01042	KNG1_HUMAN	KNG1	X	X
C4b-binding protein beta chain	P20851	C4BPB_HUMAN	C4BPB	X	X
Plasma serine protease inhibitor	P05154	IPSP_HUMAN	SERPINA5	X	X
Plasma protease C1 inhibitor	P05155	IC1_HUMAN	SERPING1	X	X
Alpha-1B-glycoprotein	P04217	A1BG_HUMAN	A1BG	X	X
Angiotensinogen	P01019	ANGT_HUMAN	AGT	X	X
Inter-alpha-trypsin inhibitor heavy chain H2	P19823	ITIH2_HUMAN	ITIH2	X	X
Immunoglobulin heavy constant gamma 2	P01859	IGHG2_HUMAN	IGHG2	X	X
Immunoglobulin heavy constant gamma 3	P01860	IGHG3_HUMAN	IGHG3	X	X
Vitronectin	P04004	VTNC_HUMAN	VTN	X	X
Immunoglobulin lambda-like polypeptide 5	B9A064	IGLL5_HUMAN	IGLL5	X	X
Glutathione peroxidase 3	P22352	GPX3_HUMAN	GPX3	X	X
Vitamin D-binding protein	P02774	VTDB_HUMAN	GC	X	X
Inter-alpha-trypsin inhibitor heavy chain H1	P19827	ITIH1_HUMAN	ITIH1	X	X
Complement C1q subcomponent subunit C	P02747	C1QC_HUMAN	C1QC	X	X
Ceruloplasmin	P00450	CERU_HUMAN	CP	X	X
Immunoglobulin kappa variable 4-1	P06312	KV401_HUMAN	IGKV4-1	X	X
Immunoglobulin kappa variable 3-20	P01619	KV320_HUMAN	IGKV3-20	X	X
Antithrombin-III	P01008	ANT3_HUMAN	SERPINC1	X	X
Hemoglobin subunit beta	P68871	HBB_HUMAN	HBB	X	X
Apolipoprotein A-I	P02647	APOA1_HUMAN	APOA1	X	X
Fibronectin	P02751	FINC_HUMAN	FN1	X	X
Complement C2	P06681	CO2_HUMAN	C2	X	X
Serum amyloid P-component	P02743	SAMP_HUMAN	APCS	X	X

Cholinesterase	P06276	CHLE_HUMAN	BCHE	X	X
Hemopexin	P02790	HEMO_HUMAN	HPX	X	X
Platelet basic protein	P02775	CXCL7_HUMAN	PPBP	X	X
Immunoglobulin heavy variable 3-13	P01766	HV313_HUMAN	IGHV3-13	X	X
Immunoglobulin heavy variable 3-15	A0A0B4J1V0	HV315_HUMAN	IGHV3-15	X	X
Apolipoprotein E	P02649	APOE_HUMAN	APOE	X	X
Immunoglobulin heavy constant alpha 1	P01876	IGHA1_HUMAN	IGHA1	X	X
Beta-Ala-His dipeptidase	Q96KN2	CNDP1_HUMAN	CNDP1	X	X
N-acetylmuramoyl-L-alanine amidase	Q96PD5	PGRP2_HUMAN	PGLYRP2	X	X
Haptoglobin	P00738	HPT_HUMAN	HPT	X	X
Immunoglobulin heavy variable 3-49	A0A0A0MS15	HV349_HUMAN	IGHV3-49	X	X
Alpha-2-macroglobulin	P01023	A2MG_HUMAN	A2M	X	X
Immunoglobulin J chain	P01591	IGJ_HUMAN	JCHAIN	X	X
Apolipoprotein C-I	P02654	APOC1_HUMAN	APOC1	X	X
CD5 antigen-like	O43866	CD5L_HUMAN	CD5L	X	X
Attractin	O75882	ATRN_HUMAN	ATRN	X	X
Apolipoprotein C-II	P02655	APOC2_HUMAN	APOC2	X	X
Vitamin K-dependent protein S	P07225	PROS_HUMAN	PROS1	X	X
Zinc-alpha-2-glycoprotein	P25311	ZA2G_HUMAN	AZGP1	X	X
Gelsolin	P06396	GELS_HUMAN	GSN	X	X
Immunoglobulin heavy constant mu	P01871	IGHM_HUMAN	IGHM	X	X
Afamin	P43652	AFAM_HUMAN	AFM	X	X
Serotransferrin	P02787	TRFE_HUMAN	TF	X	X
Insulin-like growth factor-binding protein complex acid labile subunit	P35858	ALS_HUMAN	IGFALS	X	X
Clusterin	P10909	CLUS_HUMAN	CLU	X	X
Immunoglobulin heavy variable 3-7	P01780	HV307_HUMAN	IGHV3-7	X	X
Tetranectin	P05452	TETN_HUMAN	CLEC3B	X	X
Kallistatin	P29622	KAIN_HUMAN	SERPINA4	X	X
Lumican	P51884	LUM_HUMAN	LUM	X	X
Alpha-2-antiplasmin	P08697	A2AP_HUMAN	SERPINF2	X	X
Immunoglobulin kappa variable 3-11	P04433	KV311_HUMAN	IGKV3-11	X	X
Plasminogen	P00747	PLMN_HUMAN	PLG	X	X
Thyroxine-binding globulin	P05543	THBG_HUMAN	SERPINA7	X	X

Transthyretin	P02766	TTHY_HUMAN	TTR	X	X
Carboxypeptidase N subunit 2	P22792	CPN2_HUMAN	CPN2	X	X
Ficolin-3	O75636	FCN3_HUMAN	FCN3	X	X
Adiponectin	Q15848	ADIPO_HUMAN	ADIPOQ	X	X
Complement factor H	P08603	CFAH_HUMAN	CFH	X	X
Alpha-1-antichymotrypsin	P01011	AACT_HUMAN	SERPINA3	X	X
Sex hormone-binding globulin	P04278	SHBG_HUMAN	SHBG	X	X
Albumin	P02768	ALBU_HUMAN	ALB	X	X
Complement factor B	P00751	CFAB_HUMAN	CFB	X	X
Sulfhydryl oxidase 1	O00391	QSOX1_HUMAN	QSOX1	X	X
Inter-alpha-trypsin inhibitor heavy chain H4	Q14624	ITIH4_HUMAN	ITIH4	X	X
Coagulation factor V	P12259	FA5_HUMAN	F5	X	X
Complement C4-A	P0C0L4	CO4A_HUMAN	C4A	X	X
Pigment epithelium-derived factor	P36955	PEDF_HUMAN	SERPINF1	X	X
Immunoglobulin kappa variable 2-24	A0A0C4DH68	KV224_HUMAN	IGKV2-24	X	X
Phosphatidylcholine-sterol acyltransferase	P04180	LCAT_HUMAN	LCAT	X	X
Immunoglobulin heavy variable 3-72	A0A0B4J1Y9	HV372_HUMAN	IGHV3-72	X	X
Alpha-1-antitrypsin	P01009	A1AT_HUMAN	SERPINA1	X	X
Retinol-binding protein 4	P02753	RET4_HUMAN	RBP4	X	X
Complement C1q subcomponent subunit B	P02746	C1QB_HUMAN	C1QB	X	X
C4b-binding protein alpha chain	P04003	C4BPA_HUMAN	C4BPA	X	X
Complement C1r subcomponent-like protein	Q9NZP8	C1RL_HUMAN	C1RL	X	X
Alpha-1-acid glycoprotein 1	P02763	A1AG1_HUMAN	ORM1	X	X
Complement factor I	P05156	CFAI_HUMAN	CFI	X	X
Apolipoprotein L1	O14791	APOL1_HUMAN	APOL1	X	X
Complement C4-B	P0C0L5	CO4B_HUMAN	C4B	X	X
Immunoglobulin heavy constant gamma 4	P01861	IGHG4_HUMAN	IGHG4	X	X
Apolipoprotein A-IV	P06727	APOA4_HUMAN	APOA4	X	X
Serum amyloid A-4 protein	P35542	SAA4_HUMAN	SAA4	X	X
Selenoprotein P	P49908	SEPP1_HUMAN	SELENOP	X	X
Leucine-rich alpha-2-glycoprotein	P02750	A2GL_HUMAN	LRG1	X	X
Complement C5	P01031	CO5_HUMAN	C5	X	X
Immunoglobulin kappa constant	P01834	IGKC_HUMAN	IGKC	X	X

Immunoglobulin kappa variable 1-16	P04430	KV116_HUMAN	IGKV1-16	X	X
Apolipoprotein A-II	P02652	APOA2_HUMAN	APOA2	X	X
Galectin-3-binding protein	Q08380	LG3BP_HUMAN	LGALS3BP	X	X
Alpha-2-HS-glycoprotein	P02765	FETUA_HUMAN	AHSG	X	X
Beta-2-glycoprotein 1	P02749	APOH_HUMAN	APOH	X	X
Protein Z-dependent protease inhibitor	Q9UK55	ZPI_HUMAN	SERPINA10	X	X
Complement C3	P01024	CO3_HUMAN	C3	X	X
Carboxypeptidase B2	Q96IY4	CBPB2_HUMAN	CPB2	X	X
Apolipoprotein D	P05090	APOD_HUMAN	APOD	X	X
Alpha-1-acid glycoprotein 2	P19652	A1AG2_HUMAN	ORM2	X	X
Apolipoprotein M	O95445	APOM_HUMAN	APOM	X	X
Protein AMBP	P02760	AMBP_HUMAN	AMBP	X	X
Hemoglobin subunit alpha	P69905	HBA_HUMAN	HBA1	X	X
Heparin cofactor 2	P05546	HEP2_HUMAN	SERPIND1	X	X
Apolipoprotein B-100	P04114	APOB_HUMAN	APOB	X	X
Corticosteroid-binding globulin	P08185	CBG_HUMAN	SERPINA6	X	X
Immunoglobulin lambda variable 1-51	P01701	LV151_HUMAN	IGLV1-51	X	X
Haptoglobin-related protein	P00739	HPTR_HUMAN	HPR	X	X
Prothrombin	P00734	THRB_HUMAN	F2	X	X
Phosphatidylinositol-glycan-specific phospholipase D	P80108	PHLD_HUMAN	GPLD1	X	X
Histidine-rich glycoprotein	P04196	HRG_HUMAN	HRG	X	X

Table S2. Proteins identified in the serum samples pretreated with AuNPs **(method 2)** belonging to HER2-positive BC patients that were obtained before starting the neoadjuvant treatment. These patients showed different response after the NAT treatment: responders (n = 6), non-responders (n = 4). The accession number, gene name and species (Human) were reported.

Protein Name	UniProt Name	Entry Name	Gene	Responders	Non-responders
Angiotensinogen	P01019	ANGT_HUMAN	AGT	X	
Ficolin-2	Q15485	FCN2_HUMAN	FCN2	X	
Hemoglobin subunit beta	P68871	HBB_HUMAN	HBB	X	
Immunoglobulin lambda variable 1-47	P01700	LV147_HUMAN	IGLV1-47	X	
Immunoglobulin J chain	P01591	IGJ_HUMAN	JCHAIN	X	
Hemoglobin subunit alpha	P69905	HBA_HUMAN	HBA1	X	
Pregnancy zone protein	P20742	PZP_HUMAN	PZP		X
Complement component C8 gamma chain	P07360	CO8G_HUMAN	C8G		X
Carboxypeptidase N catalytic chain	P15169	CBPN_HUMAN	CPN1		X
C4b-binding protein beta chain	P20851	C4BPB_HUMAN	C4BPB		X
Complement C1s subcomponent	P09871	C1S_HUMAN	C1S		X
Complement C1r subcomponent	P00736	C1R_HUMAN	C1R		X
Mannan-binding lectin serine protease 1	P48740	MASP1_HUMAN	MASP1		X
Coagulation factor XII	P00748	FA12_HUMAN	F12		X
Immunoglobulin lambda-like polypeptide 5	B9A064	IGLL5_HUMAN	IGLL5		X
Extracellular matrix protein 1	Q16610	ECM1_HUMAN	ECM1		X
Immunoglobulin kappa variable 4-1	P06312	KV401_HUMAN	IGKV4-1		X
Plasma kallikrein	P03952	KLKB1_HUMAN	KLKB1		X
Hyaluronan-binding protein 2	Q14520	HABP2_HUMAN	HABP2		X
Complement component C8 beta chain	P07358	CO8B_HUMAN	C8B		X
Complement C1q subcomponent subunit A	P02745	C1QA_HUMAN	C1QA		X
Complement component C7	P10643	CO7_HUMAN	C7		X
Vitamin K-dependent protein C	P04070	PROC_HUMAN	PROC		X
Coagulation factor X	P00742	FA10_HUMAN	F10		X
CD5 antigen-like	O43866	CD5L_HUMAN	CD5L		X
Attractin	O75882	ATRN_HUMAN	ATRN		X
Complement component C8 alpha chain	P07357	CO8A_HUMAN	C8A		X
Afamin	P43652	AFAM_HUMAN	AFM		X
Insulin-like growth factor-binding protein complex acid labile subunit	P35858	ALS_HUMAN	IGFALS		X
Immunoglobulin heavy variable 3-7	P01780	HV307_HUMAN	IGHV3-7		X
Immunoglobulin kappa variable 3-11	P04433	KV311_HUMAN	IGKV3-11		X
Plasminogen	P00747	PLMN_HUMAN	PLG		X
Coagulation factor XIII B chain	P05160	F13B_HUMAN	F13B		X
Ficolin-3	O75636	FCN3_HUMAN	FCN3		X
Complement factor H	P08603	CFAH_HUMAN	CFH		X
Sex hormone-binding globulin	P04278	SHBG_HUMAN	SHBG		X

Complement factor H-related protein 1	Q03591	FHR1_HUMAN	CFHR1		X
C4b-binding protein alpha chain	P04003	C4BPA_HUMAN	C4BPA		X
Complement component C9	P02748	CO9_HUMAN	C9		X
Complement factor H-related protein 2	P36980	FHR2_HUMAN	CFHR2		X
Immunoglobulin lambda constant 3	P0DOY3	IGLC3_HUMAN	IGLC3		X
Complement factor I	P05156	CFAI_HUMAN	CFI		X
Complement C1r subcomponent-like protein	Q9NZP8	C1RL_HUMAN	C1RL		X
Selenoprotein P	P49908	SEPP1_HUMAN	SELENOP		X
Complement C5	P01031	CO5_HUMAN	C5		X
Apolipoprotein A-II	P02652	APOA2_HUMAN	APOA2		X
Apolipoprotein D	P05090	APOD_HUMAN	APOD		X
Keratin, type I cytoskeletal 9	P35527	K1C9_HUMAN	KRT9		X
Prothrombin	P00734	THRB_HUMAN	F2		X
Phosphatidylinositol-glycan-specific phospholipase D	P80108	PHLD_HUMAN	GPLD1		X
Fetuin-B	Q9UGM5	FETUB_HUMAN	FETUB		X
Apolipoprotein C-III	P02656	APOC3_HUMAN	APOC3	X	X
Serum paraoxonase/ arylesterase 1	P27169	PON1_HUMAN	PON1	X	X
Immunoglobulin heavy constant gamma 1	P01857	IGHG1_HUMAN	IGHG1	X	X
Inter-alpha-trypsin inhibitor heavy chain H3	Q06033	ITIH3_HUMAN	ITIH3	X	X
Kininogen-1	P01042	KNG1_HUMAN	KNG1	X	X
Plasma protease C1 inhibitor	P05155	IC1_HUMAN	SERPING1	X	X
Alpha-1B-glycoprotein	P04217	A1BG_HUMAN	A1BG	X	X
Inter-alpha-trypsin inhibitor heavy chain H2	P19823	ITIH2_HUMAN	ITIH2	X	X
Immunoglobulin heavy constant gamma 2	P01859	IGHG2_HUMAN	IGHG2	X	X
Vitronectin	P04004	VTNC_HUMAN	VTN	X	X
Keratin, type II cytoskeletal 1	P04264	K2C1_HUMAN	KRT1	X	X
Inter-alpha-trypsin inhibitor heavy chain H1	P19827	ITIH1_HUMAN	ITIH1	X	X
Vitamin D-binding protein	P02774	VTDB_HUMAN	GC	X	X
Complement C1q subcomponent subunit C	P02747	C1QC_HUMAN	C1QC	X	X
Ceruloplasmin	P00450	CERU_HUMAN	CP	X	X
Antithrombin-III	P01008	ANT3_HUMAN	SERPINC1	X	X
Apolipoprotein A-I	P02647	APOA1_HUMAN	APOA1	X	X
Fibronectin	P02751	FINC_HUMAN	FN1	X	X
Complement C2	P06681	CO2_HUMAN	C2	X	X
Hemopexin	P02790	HEMO_HUMAN	HPX	X	X
Apolipoprotein E	P02649	APOE_HUMAN	APOE	X	X
Immunoglobulin heavy constant alpha 1	P01876	IGHA1_HUMAN	IGHA1	X	X
N-acetylmuramoyl-L-alanine amidase	Q96PD5	PGRP2_HUMAN	PGLYRP2	X	X
Haptoglobin	P00738	HPT_HUMAN	HPT	X	X
Alpha-2-macroglobulin	P01023	A2MG_HUMAN	A2M	X	X
Vitamin K-dependent protein S	P07225	PROS_HUMAN	PROS1	X	X

Gelsolin	P06396	GELS_HUMAN	GSN	X	X
Immunoglobulin heavy constant mu	P01871	IGHM_HUMAN	IGHM	X	X
Serotransferrin	P02787	TRFE_HUMAN	TF	X	X
Clusterin	P10909	CLUS_HUMAN	CLU	X	X
Lumican	P51884	LUM_HUMAN	LUM	X	X
Alpha-2-antiplasmin	P08697	A2AP_HUMAN	SERPINF2	X	X
Carboxypeptidase N subunit 2	P22792	CPN2_HUMAN	CPN2	X	X
Alpha-1-antichymotrypsin	P01011	AACT_HUMAN	SERPINA3	X	X
Albumin	P02768	ALBU_HUMAN	ALB	X	X
Complement factor B	P00751	CFAB_HUMAN	CFB	X	X
Inter-alpha-trypsin inhibitor heavy chain H4	Q14624	ITIH4_HUMAN	ITIH4	X	X
Complement C4-A	P0C0L4	CO4A_HUMAN	C4A	X	X
Alpha-1-antitrypsin	P01009	A1AT_HUMAN	SERPINA1	X	X
Retinol-binding protein 4	P02753	RET4_HUMAN	RBP4	X	X
Complement C1q subcomponent subunit B	P02746	C1QB_HUMAN	C1QB	X	X
Apolipoprotein L1	O14791	APOL1_HUMAN	APOL1	X	X
Complement C4-B	P0C0L5	CO4B_HUMAN	C4B	X	X
Apolipoprotein A-IV	P06727	APOA4_HUMAN	APOA4	X	X
Immunoglobulin kappa constant	P01834	IGKC_HUMAN	IGKC	X	X
Alpha-2-HS-glycoprotein	P02765	FETUA_HUMAN	AHSG	X	X
Beta-2-glycoprotein 1	P02749	APOH_HUMAN	APOH	X	X
Complement C3	P01024	CO3_HUMAN	C3	X	X
Apolipoprotein M	O95445	APOM_HUMAN	APOM	X	X
Protein AMBP	P02760	AMBP_HUMAN	AMBP	X	X
Keratin, type I cytoskeletal 10	P13645	K1C10_HUMAN	KRT10	X	X
Heparin cofactor 2	P05546	HEP2_HUMAN	SERPIND1	X	X
Apolipoprotein B-100	P04114	APOB_HUMAN	APOB	X	X
Haptoglobin-related protein	P00739	HPTR_HUMAN	HPR	X	X
Histidine-rich glycoprotein	P04196	HRG_HUMAN	HRG	X	X

Table S3. Proteins identified in the serum samples pretreated with PtNPs **(method 3)** belonging to HER2-positive BC patients that were obtained before starting the neoadjuvant treatment. These patients showed different response after the NAT treatment: responders (*n* = 6), non-responders (*n* = 4). The accession number, gene name and species (Human) were reported.

Protein Name	UniProt Name	Entry Name	Gene	Responders	Non-responders
Apolipoprotein C-III	P02656	APOC3_HUMAN	APOC3	X	
Gelsolin	P06396	GELS_HUMAN	GSN	X	
Immunoglobulin kappa constant	P01834	IGKC_HUMAN	IGKC	X	
Apolipoprotein A-II	P02652	APOA2_HUMAN	APOA2	X	
Galectin-3-binding protein	Q08380	LG3BP_HUMAN	LGALS3BP	X	
Immunoglobulin kappa variable 2D-28	P01615	KVD28_HUMAN	IGKV2D-28	X	
Plasminogen	P00747	PLMN_HUMAN	PLG		X
Complement component C8 gamma chain	P07360	CO8G_HUMAN	C8G		X
Complement factor H-related protein 1	Q03591	FHR1_HUMAN	CFHR1		X
Immunoglobulin lambda-like polypeptide 5	B9A064	IGLL5_HUMAN	IGLL5		X
Alpha-1-antitrypsin	P01009	A1AT_HUMAN	SERPINA1		X
Keratin, type II cytoskeletal 1	P04264	K2C1_HUMAN	KRT1		X
Complement component C8 beta chain	P07358	CO8B_HUMAN	C8B		X
Carboxypeptidase B2	Q96IY4	CBPB2_HUMAN	CPB2		X
Apolipoprotein D	P05090	APOD_HUMAN	APOD		X
Immunoglobulin J chain	P01591	IGJ_HUMAN	JCHAIN		X
Haptoglobin-related protein	P00739	HPTR_HUMAN	HPR		X
Serum paraoxonase/ arylesterase 1	P27169	PON1_HUMAN	PON1	X	X
Immunoglobulin heavy constant gamma 1	P01857	IGHG1_HUMAN	IGHG1	X	X
Inter-alpha-trypsin inhibitor heavy chain H3	Q06033	ITIH3_HUMAN	ITIH3	X	X
Carboxypeptidase N subunit 2	P22792	CPN2_HUMAN	CPN2	X	X
Ficolin-3	O75636	FCN3_HUMAN	FCN3	X	X
Complement factor H	P08603	CFAH_HUMAN	CFH	X	X
Kininogen-1	P01042	KNG1_HUMAN	KNG1	X	X
Plasma protease C1 inhibitor	P05155	IC1_HUMAN	SERPING1	X	X
Albumin	P02768	ALBU_HUMAN	ALB	X	X
Inter-alpha-trypsin inhibitor heavy chain H2	P19823	ITIH2_HUMAN	ITIH2	X	X
Complement factor B	P00751	CFAB_HUMAN	CFB	X	X
Inter-alpha-trypsin inhibitor heavy chain H4	Q14624	ITIH4_HUMAN	ITIH4	X	X
Vitronectin	P04004	VTNC_HUMAN	VTN	X	X
Retinol-binding protein 4	P02753	RET4_HUMAN	RBP4	X	X
Complement C1q subcomponent subunit B	P02746	C1QB_HUMAN	C1QB	X	X
Inter-alpha-trypsin inhibitor heavy chain H1	P19827	ITIH1_HUMAN	ITIH1	X	X

Vitamin D-binding protein	P02774	VTDB_HUMAN	GC	X	X
C4b-binding protein alpha chain	P04003	C4BPA_HUMAN	C4BPA	X	X
Complement C1q subcomponent subunit C	P02747	C1QC_HUMAN	C1QC	X	X
Complement component C9	P02748	CO9_HUMAN	C9	X	X
Immunoglobulin kappa variable 3-20	P01619	KV320_HUMAN	IGKV3-20	X	X
Antithrombin-III	P01008	ANT3_HUMAN	SERPINC1	X	X
Complement factor I	P05156	CFAI_HUMAN	CFI	X	X
Apolipoprotein A-I	P02647	APOA1_HUMAN	APOA1	X	X
Fibronectin	P02751	FINC_HUMAN	FN1	X	X
Complement C4-B	P0C0L5	CO4B_HUMAN	C4B	X	X
Complement C2	P06681	CO2_HUMAN	C2	X	X
Apolipoprotein A-IV	P06727	APOA4_HUMAN	APOA4	X	X
Hemopexin	P02790	HEMO_HUMAN	HPX	X	X
Complement C5	P01031	CO5_HUMAN	C5	X	X
Apolipoprotein E	P02649	APOE_HUMAN	APOE	X	X
Beta-2-glycoprotein 1	P02749	APOH_HUMAN	APOH	X	X
Alpha-2-HS-glycoprotein	P02765	FETUA_HUMAN	AHSG	X	X
Immunoglobulin heavy constant alpha 1	P01876	IGHA1_HUMAN	IGHA1	X	X
Complement C3	P01024	CO3_HUMAN	C3	X	X
N-acetylmuramoyl-L-alanine amidase	Q96PD5	PGRP2_HUMAN	PGLYRP2	X	X
Haptoglobin	P00738	HPT_HUMAN	HPT	X	X
Alpha-2-macroglobulin	P01023	A2MG_HUMAN	A2M	X	X
Apolipoprotein M	O95445	APOM_HUMAN	APOM	X	X
CD5 antigen-like	O43866	CD5L_HUMAN	CD5L	X	X
Protein AMBP	P02760	AMBP_HUMAN	AMBP	X	X
Vitamin K-dependent protein S	P07225	PROS_HUMAN	PROS1	X	X
Immunoglobulin heavy constant mu	P01871	IGHM_HUMAN	IGHM	X	X
Afamin	P43652	AFAM_HUMAN	AFM	X	X
Apolipoprotein B-100	P04114	APOB_HUMAN	APOB	X	X
Serotransferrin	P02787	TRFE_HUMAN	TF	X	X
Clusterin	P10909	CLUS_HUMAN	CLU	X	X
Prothrombin	P00734	THRB_HUMAN	F2	X	X
Histidine-rich glycoprotein	P04196	HRG_HUMAN	HRG	X	X
Alpha-2-antiplasmin	P08697	A2AP_HUMAN	SERPINF2	X	X

Table S4. List of 43 common proteins identified in the serum of responders pretreated by the three different methods (with and without NPs). The accession number, gene name and species (Human) were reported.

Protein Name	UniProt Name	Entry Name	Gene
Apolipoprotein C-III	P02656	APOC3_HUMAN	APOC3
Serum paraoxonase/ arylesterase 1	P27169	PON1_HUMAN	PON1
Immunoglobulin heavy constant gamma 1	P01857	IGHG1_HUMAN	IGHG1
Inter-alpha-trypsin inhibitor heavy chain H3	Q06033	ITIH3_HUMAN	ITIH3
Kininogen-1	P01042	KNG1_HUMAN	KNG1
Plasma protease C1 inhibitor	P05155	IC1_HUMAN	SERPING1
Inter-alpha-trypsin inhibitor heavy chain H2	P19823	ITIH2_HUMAN	ITIH2
Vitronectin	P04004	VTNC_HUMAN	VTN
Vitamin D-binding protein	P02774	VTDB_HUMAN	GC
Inter-alpha-trypsin inhibitor heavy chain H1	P19827	ITIH1_HUMAN	ITIH1
Complement C1q subcomponent subunit C	P02747	C1QC_HUMAN	C1QC
Antithrombin-III	P01008	ANT3_HUMAN	SERPINC1
Fibronectin	P02751	FINC_HUMAN	FN1
Apolipoprotein A-I	P02647	APOA1_HUMAN	APOA1
Complement C2	P06681	CO2_HUMAN	C2
Hemopexin	P02790	HEMO_HUMAN	HPX
Apolipoprotein E	P02649	APOE_HUMAN	APOE
Immunoglobulin heavy constant alpha 1	P01876	IGHA1_HUMAN	IGHA1
N-acetylmuramoyl-L-alanine amidase	Q96PD5	PGRP2_HUMAN	PGLYRP2
Haptoglobin	P00738	HPT_HUMAN	HPT
Alpha-2-macroglobulin	P01023	A2MG_HUMAN	A2M
Vitamin K-dependent protein S	P07225	PROS_HUMAN	PROS1
Gelsolin	P06396	GELS_HUMAN	GSN
Immunoglobulin heavy constant mu	P01871	IGHM_HUMAN	IGHM
Serotransferrin	P02787	TRFE_HUMAN	TF
Clusterin	P10909	CLUS_HUMAN	CLU
Alpha-2-antiplasmin	P08697	A2AP_HUMAN	SERPINF2
Carboxypeptidase N subunit 2	P22792	CPN2_HUMAN	CPN2
Albumin	P02768	ALBU_HUMAN	ALB
Complement factor B	P00751	CFAB_HUMAN	CFB
Inter-alpha-trypsin inhibitor heavy chain H4	Q14624	ITIH4_HUMAN	ITIH4
Retinol-binding protein 4	P02753	RET4_HUMAN	RBP4
Complement C1q subcomponent subunit B	P02746	C1QB_HUMAN	C1QB
Complement C4-B	P0C0L5	CO4B_HUMAN	C4B
Apolipoprotein A-IV	P06727	APOA4_HUMAN	APOA4
Immunoglobulin kappa constant	P01834	IGKC_HUMAN	IGKC
Alpha-2-HS-glycoprotein	P02765	FETUA_HUMAN	AHSG
Beta-2-glycoprotein 1	P02749	APOH_HUMAN	APOH
Complement C3	P01024	CO3_HUMAN	C3
Apolipoprotein M	O95445	APOM_HUMAN	APOM
Protein AMBP	P02760	AMBP_HUMAN	AMBP
Apolipoprotein B-100	P04114	APOB_HUMAN	APOB
Histidine-rich glycoprotein	P04196	HRG_HUMAN	HRG

Table S5. List of 54 common proteins identified in the serum of non-responders pretreated by the three different methods (with and without NPs). The accession number, gene name and species (Human) were reported.

Protein Name	UniProt Name	Entry Name	Gene
Serum paraoxonase/ arylesterase 1	P27169	PON1_HUMAN	PON1
Immunoglobulin heavy constant gamma 1	P01857	IGHG1_HUMAN	IGHG1
Inter-alpha-trypsin inhibitor heavy chain H3	Q06033	ITIH3_HUMAN	ITIH3
Kininogen-1	P01042	KNG1_HUMAN	KNG1
cPlasma protease C1 inhibitor	P05155	IC1_HUMAN	SERPING1

Inter-alpha-trypsin inhibitor heavy chain H2	P19823	ITIH2_HUMAN	ITIH2
Vitronectin	P04004	VTNC_HUMAN	VTN
Immunoglobulin lambda-like polypeptide 5	B9A064	IGLL5_HUMAN	IGLL5
Inter-alpha-trypsin inhibitor heavy chain H1	P19827	ITIH1_HUMAN	ITIH1
Vitamin D-binding protein	P02774	VTDB_HUMAN	GC
Complement C1q subcomponent subunit C	P02747	C1QC_HUMAN	C1QC
Antithrombin-III	P01008	ANT3_HUMAN	SERPINC1
Apolipoprotein A-I	P02647	APOA1_HUMAN	APOA1
Fibronectin	P02751	FINC_HUMAN	FN1
Complement C2	P06681	CO2_HUMAN	C2
Hemopexin	P02790	HEMO_HUMAN	HPX
Apolipoprotein E	P02649	APOE_HUMAN	APOE
Immunoglobulin heavy constant alpha 1	P01876	IGHA1_HUMAN	IGHA1
N-acetylmuramoyl-L-alanine amidase	Q96PD5	PGRP2_HUMAN	PGLYRP2
Haptoglobin	P00738	HPT_HUMAN	HPT
Alpha-2-macroglobulin	P01023	A2MG_HUMAN	A2M
CD5 antigen-like	O43866	CD5L_HUMAN	CD5L
Vitamin K-dependent protein S	P07225	PROS_HUMAN	PROS1
Immunoglobulin heavy constant mu	P01871	IGHM_HUMAN	IGHM
Afamin	P43652	AFAM_HUMAN	AFM
Serotransferrin	P02787	TRFE_HUMAN	TF
Clusterin	P10909	CLUS_HUMAN	CLU
Alpha-2-antiplasmin	P08697	A2AP_HUMAN	SERPINF2
Plasminogen	P00747	PLMN_HUMAN	PLG
Carboxypeptidase N subunit 2	P22792	CPN2_HUMAN	CPN2
Ficolin-3	O75636	FCN3_HUMAN	FCN3
Complement factor H	P08603	CFAH_HUMAN	CFH
Albumin	P02768	ALBU_HUMAN	ALB
Complement factor H-related protein 1	Q03591	FHR1_HUMAN	CFHR1
Complement factor B	P00751	CFAB_HUMAN	CFB
Inter-alpha-trypsin inhibitor heavy chain H4	Q14624	ITIH4_HUMAN	ITIH4
Alpha-1-antitrypsin	P01009	A1AT_HUMAN	SERPINA1
Retinol-binding protein 4	P02753	RET4_HUMAN	RBP4
Complement C1q subcomponent subunit B	P02746	C1QB_HUMAN	C1QB
C4b-binding protein alpha chain	P04003	C4BPA_HUMAN	C4BPA
Complement factor I	P05156	CFAI_HUMAN	CFI
Complement C4-B	P0C0L5	CO4B_HUMAN	C4B
Apolipoprotein A-IV	P06727	APOA4_HUMAN	APOA4
Complement C5	P01031	CO5_HUMAN	C5
Alpha-2-HS-glycoprotein	P02765	FETUA_HUMAN	AHSG
Beta-2-glycoprotein 1	P02749	APOH_HUMAN	APOH
Complement C3	P01024	CO3_HUMAN	C3
Apolipoprotein D	P05090	APOD_HUMAN	APOD
Apolipoprotein M	O95445	APOM_HUMAN	APOM
Protein AMBP	P02760	AMBP_HUMAN	AMBP
Apolipoprotein B-100	P04114	APOB_HUMAN	APOB
Haptoglobin-related protein	P00739	HPTR_HUMAN	HPR
Prothrombin	P00734	THRB_HUMAN	F2
Histidine-rich glycoprotein	P04196	HRG_HUMAN	HRG

Table S6. List of the upregulated and downregulated proteins (a fold change ≥ 1.5 and P < 0.05) found after the TMT labeling-based quantitative proteomic analysis of 6 serum samples from primary HER2-positive breast cancer cases, including 3 trastuzumab-based therapy-resistant and 3 trastuzumab-based therapy responsive cases developed by T. Yang et al. [46].

Uniprot Code	Gene name	Protein Name	*p*-Value	Response to NAC
P04114	APOB	Apolipoprotein B-100	0.0019	↑ Responders
Q14624	ITH4	Inter-alpha-trypsin inhibitor heavy chain H4	0.0196	↑ Responders
P06727	APOA4	Apolipoprotein A-IV	0.0047	↓ Responders
P02748	C9	Complement component C9	0.0053	↑ Responders
Q06033	ITIH3	Inter-alpha-trypsin inhibitor heavy chain H3	0.0439	↑ Responders
P48740	MASP1	Mannan-binding lectin serine protease 1	0.0128	↑ Responders
P08571	CD14	Monocyte differentiation antigen CD14	0.0021	↑ Responders
P13796	LCP1	Plastin-2	0.0042	↑ Responders
P01034	CST3	Cystatin-C	0.0010	↑ Responders
P18428	LBP	Lipopolysaccharide-binding protein	0.0183	↑ Responders
P07195	LDHB	L-lactate dehydrogenase B chain	0.0325	↓ Responders
O00187	MASP2	Mannan-binding lectin serine protease 2	0.0146	↑ Responders
P00338	LDHA	L-lactate dehydrogenase A chain	0.0179	↓ Responders
P10124	SRGN	Serglycin	0.0013	↑ Responders
P08195	SLC3A2	4F2 cell-surface antigen heavy chain	0.0002	↑ Responders
Q8NFT6	DBF4B	Protein DBF4 homolog B	0.0059	↑ Responders
P11766	ADH5	Alcohol dehydrogenase class-3	0.0208	↓ Responders
P13693	TPT1	Translationally-controlled tumor protein	0.0430	↓ Responders
